# ﻿Centipedes (Myriapoda, Chilopoda) of Aldabra Atoll (Seychelles)

**DOI:** 10.3897/zookeys.1228.143007

**Published:** 2025-02-21

**Authors:** George Popovici, Gregory D. Edgecombe

**Affiliations:** 1 Department of Life Sciences, Imperial College London, London SW7 2AZ, UK Department of Life Sciences, Imperial College London London United Kingdom; 2 "Grigore Antipa" National Museum of Natural History, Pavel Dimitrievici Kiseleff St. 1, 011341, Bucharest, Romania “Grigore Antipa” National Museum of Natural History Bucharest Romania; 3 The Natural History Museum, Cromwell Road, London SW7 5BD, UK The Natural History Museum London United Kingdom

**Keywords:** Checklist, taxonomy, Western Indian Ocean

## Abstract

Centipedes collected during Royal Society surveys of the arthropod fauna of the Aldabra Atoll in 1968–1975 are identified, described, and illustrated to provide the first checklist to the Aldabran centipede fauna, comprising 12 species. These newly include the lithobiomorph *Lamyctestristani* (Pocock, 1893), the scolopendromorphs *Scolopendramorsitans*Linnaeus 1758, Cryptopscf.japonicus Takakuwa, 1934, *Cryptopsmauritianus* Verhoeff, 1939, and *Cryptopsnigropictus* Takakuwa, 1936, and the geophilomorphs Ityphiluscf.taeniaformis (Lawrence, 1960), *Mecistocephalusangusticeps* (Ribaut, 1914), *Mecistocephaluslohmanderi* Verhoeff, 1939, *Orphnaeusdekanius* Verhoeff, 1938, Ribautiacf.paucipes Attems, 1952, and *Tuobasydneyensis* (Pocock, 1891). The geophilomorph genera *Hovanyx* Lawrence, 1960, **syn. nov.**, and *Mixophilus* Silvestri, 1929, **syn. nov.**, are revised in light of the examined material and hereby designated junior subjective synonyms of *Tuoba* Chamberlin, 1920 with the species *Geophiluslemuricus* Verhoeff, 1939, **syn. nov.**, and *Hovanyxwaterloti* Lawrence, 1960, **syn. nov.**, designated as junior subjective synonyms of *T.sydneyensis*. The oryid genus *Nycternyssa* Crabill, 1959, **syn. nov.**, is revised and designated a junior subjective synonym of *Orphnaeus* Meinert, 1870. New data on intraspecific morphological variation are presented for *C.nigropictus*, with the validity of *Cryptopsdaszaki* Lewis, 2002 being questioned following examination of its type material. The affinities and possible origins of the Aldabran centipede fauna are found to be mainly East African, with several species occurring across other islands in the Western Indian Ocean.

## ﻿Introduction

The Western Indian Ocean Islands, delimited at the north by Socotra and at the south by Madagascar, have been identified as a global biodiversity hotspot (Myers et al. 2000; Attorre and Van Damme 2020; Agnarsson et al. 2015). Although extremely variable in size, and geological origin and history, these islands harbour a terrestrial fauna notable for striking patterns of radiation and endemicity (Legros et al. 2020; Bergsten and Biström 2022; Sherlock et al. 2024). Despite being less intensively studied than their marine faunas, especially in smaller island groups, the terrestrial faunas of the Western Indian Ocean face significant risk of extinction due to the impacts of introduced species (Gerlach 2006; Griffiths and Florens 2006; Cheke 2010; Hawlitschek et al. 2011) and habitat degradation (Haverkamp et al. 2017; Ibouroi et al. 2021). Smaller island groups can offer unique insights into understanding the patterns of colonisation and diversification in an area extensively shaped by eustatic changes (Camoin et al. 2004). The paleogeographic history of coral atolls in the Western Indian Ocean in particular has identified the present composition of terrestrial faunas on these islands as recent in origin (Austin et al. 2004; Agnarsson and Kuntner 2012; Steibl et al. 2024) and reflective of multiple colonisations from mainland populations and refugia (Nagy et al. 2003; Kehlmaier et al. 2023).

Aldabra Atoll is located in the Western Indian Ocean and has an area of approximately 155 km^2^ and a maximum elevation of 8 m, being the second largest coral atoll in the world (Plummer 1995). It is composed of four main islands, Grand Terre (114 km^2^), Malabar (26.5 km^2^), Picard (9.28 km^2^), and Polymnie (1.93 km^2^), in addition to 40 small islets. The nearest large landmasses include the coast of Tanzania (640 km), the northern coast of Madagascar (425 km), Ngazidja (Comoros) (380 km), and Mahé (Seychelles) (1130 km). Palaeogeographical research on Aldabra has identified a complete submergence event during the last interglacial period, with any terrestrial fauna that had previously colonized the atoll having almost certainly disappeared approximately 125,000 years before the present (Warren et al. 2005). Re-emergence of a more permanent dry land area, together with signs of a terrestrial fauna including land snails is estimated to have occurred 118,000–80,000 years before the present, with continued fluctuations in the sea level exposing a dry land area twice as large as the current one ~ 27,000 years before the present. This was followed by dry land area reduction to its present condition after flooding of the inner lagoon ~ 5000 years before the present (Braithwaite et al. 1973; Hume et al. 2018). The faunal affinities of the Aldabra Atoll have been identified as predominantly East African, with general biogeographical patterns indicating dispersal and colonisation from continental East Africa, Madagascar, and the Seychelles (Cogan et al. 1971; Hill and Newbery 1982; Lawrence 2015), in contrast to the Mascarenes and Comoros, in which dispersal from Madagascar seemingly predominates (Agnarsson and Kuntner 2012). Given its young geological age and recent complete submergence, the fauna of Aldabra is comparatively less rich in endemic taxa than the granitic inner islands of the Seychelles (Agnarsson and Kuntner 2012).

Previous surveys of the centipede fauna of the Seychelles (Bonato and Minelli 2010; Lewis 2010b; Stoev and Gerlach 2010) have identified three species from Aldabra, *Australobiusinflatitarsis* Eason, 1978, *Mecistocephalusangusticeps* (Ribaut, 1914), and *Tuobasydneyensis* (Pocock, 1891). The threat level faced by endemic Myriapoda within the inner and outer islands of the Seychelles, including the Aldabra group, is significantly higher than the threat level faced by indigenous non-endemic (i.e., widespread) or introduced myriapods (Gerlach 2012). Observations on the natural history of large Scolopendromorpha in Mauritius and the Rodrigues provide circumstantial evidence on the vulnerability of island centipede populations to introduced predators (Lewis et al. 2010; Tercel et al. 2024).

We provide a comprehensive checklist and taxonomic revision of the centipedes from the Aldabra Atoll collected between 1968 and 1975 to provide historic baseline data to inform future conservation efforts of Aldabran terrestrial arthropods and shed light on the poorly known centipede fauna of the Western Indian Ocean.

## ﻿Materials and methods

### ﻿Morphological examination

Specimens were examined under a Nikon SMZ1270 stereomicroscope and a Leica DMR binocular microscope. Partial dissection was carried out as necessary according to the protocol outlined by Pereira (2000), with anatomical structures temporarily mounted in glycerol. Where necessary, specimens were cleared by temporary mounting in 80% lactic acid. Drawings were prepared with the aid of a camera lucida.

### ﻿Specimen data

All specimens examined are part of the Myriapoda collection of the Natural History Museum, London (**NHMUK**). Collection data for a total of 181 specimens from the Aldabra Atoll are given in the Results section for each species. Sampling was undertaken in 1974 and 1975, apart from four specimens collected in 1968 and three from 1973. In addition to material collected from Aldabra, the following specimens have been examined, either as conspecifics from other geographic regions or for comparison to Aldabra samples:

#### ﻿*Lamyctestristani* (Pocock, 1893)

NHMUK015626352, 2♀♀, Downtown, Diego Garcia, Chagos Archipelago, 7.263°S, 72.374°E, 26.06.2022, leg. W. Rabitsch, suction sampler; NHMUK015619670, 1♀, Diego Garcia, Chagos Archipelago, wetland site, 7.310°S, 72.419°E, 22.06.2022, leg. W. Rabitsch, suction sampler. NHMUK015087793, syntypes, Tristan da Cunha.

#### ﻿*Ballophilusmaldivensis* Pocock, 1906

BMNH #200555, Chilo 1952-.12.11.102, 1 ♀ (holotype), Midu (މީދޫ), Addu Atoll, Maldives, 1899–1900, Maldive-Laccadive Expedition 1899–1900.

#### ﻿*Cryptopsdaszaki* Lewis, 2002

1♂ (holotype, Spm. 1), Île aux Aigrettes, damp soil against wall of Warden’s House, 19 October 1995, leg. S. J. Lewis. Spm 4 and 5, in soil under ebony litter, grid square E4, 18.10.1995, leg. J. G. E. Lewis.

#### ﻿*Cryptopsniloticus* Lewis, 1967

BMNH(E)#200011 Chilo.1966.9.6.2, 1♀ (holotype), Blue Nile Bridge, Khartoum, Sudan, 28.09.1964, leg. J. G. E. Lewis, 1♂ (allotype), under stone, top of Nile Bank, Blue Nile Bridge, Khartoum, Sudan, 09.11.1962, leg. J. G. E. Lewis, BMNH(E) #200014 Chilo.1966.9.6.11, 1 juvenile (paratype), Blue Nile Bridge, Khartoum, Sudan, 09.11.1962.

#### ﻿*Mecistocephalusglabridorsalis* Attems, 1900

NHMUK015991408, 2 specimens, Serpent Island, Mauritius, 04.11.1995, leg. P. Daszak & J. Cottingham.

#### ﻿*Orphnaeusbrevilabiatus* (Newport, 1845)

NHMUK015991423, 1♀, S. (South) Tenasserim (Great Tenasserim River, Myanmar), BM1889.7.15.73–4, leg. E. W. Oates; NHMUK015991422, 1♂, Rangoon (Yangon, Myanmar), BM1889.7.15.73–4, leg. E. W. Oates; NHMUK015991420, 1♀, Singapore, 18.10.1898, 98.10.18.53–55, leg. H. N. Hidley; NHMUK015991421, 1♀, Takhamen, Siam (Tak province, Thailand), 1897.9.7.34, leg. S. S. Flower; NHMUK015991412, 1♂2♀♀, Agraky Hills, Yemen, leg. G. W. Berry.

#### ﻿*Orphnaeusdekanius* Verhoeff, 1938

NHMUK015991413, 1♀, Ratnapura, Ceylon (Ratnapura, Sri Lanka), 19.08.1892, 92.8.19.6; NHMUK015991414, 1♂, Pundulaya, Ceylon (Pundaluoya, Sri Lanka), 13.12.1899, 1899.12.13.42; NHMUK015991415, 1♀, Nundulaya, Ceylon (Nuwara Eliya, Sri Lanka), 13.12.1899, 99.12.13.41; NHMUK015991416, 1♀, Ceylon (Sri Lanka), 88.55; NHMUK015991417, 2♂♂1♀, Singapore, 18.10.1898, 98.10.18.53–55, leg. H. N. Hidley; NHMUK015991418, 1♂1♀, Singapore, leg. H. N. Hidley; NHMUK015991419, 1♂, Kenurus, Maldives, 1951.12.11.100, Maldive-Laccadive Expedition 1899–1900.

#### ﻿Ribautiacf.paucipes Attems, 1952

NHMUK015991409, 1 specimen, fine soil overhanging rock surface, Coin de Mire, Mauritius, 19°56.5'S, 57°37'E, 27.10.1995, leg. J. G. E. Lewis & S. J. Lewis.

#### ﻿*Tuobasydneyensis* (Pocock, 1891)

NHMUK015991411, 3♂♂3♀♀, under slabs of tuff rock, Serpent Island, Mauritius, 04.11.1995, P. Daszak & J. Cottingham.

## ﻿Results

### ﻿Checklist

**Order Lithobiomorpha**

**Family Henicopidae**

**Genus *Lamyctes* Meinert, 1868**

#### ﻿ Lamyctes tristani

Taxon classificationAnimaliaLithobiomorphaHenicopidae

(Pocock, 1893)

18A70C29-CAA0-5FD8-88E2-15C1A621B2A3

Fig. 1

##### Examined material.

41 specimens: NHMUK015991449, 1♀, Cinq Cases, 16.03.1974; NHMUK015991450, 2♀, Malabar, 14.02.1975; NHMUK015991451 1♀, Picard, 28.02.1975; NHMUK015991452, 3♀, 1 juvenile, Cinq Cases B. F., 20.03.1974; NHMUK015991453, 2♀, Picard, 23.02.1974; NHMUK015991454, 2♀, Cinq Cases, 24.03.1974; NHMUK015991458, 3♀, Malabar, 08.06.1974; NHMUK015991455, 1♀, 1 juvenile, Aldabra, 30.01.1975; NHMUK015991456, 21♀, Picard, Summer 1975, leg. V. W. Spaull; NHMUK015991457, 2♀, 1 juvenile, Ochna litter, Cinq Cases B. F., 23.03.1974.

##### Remarks.

**Figure 1.**
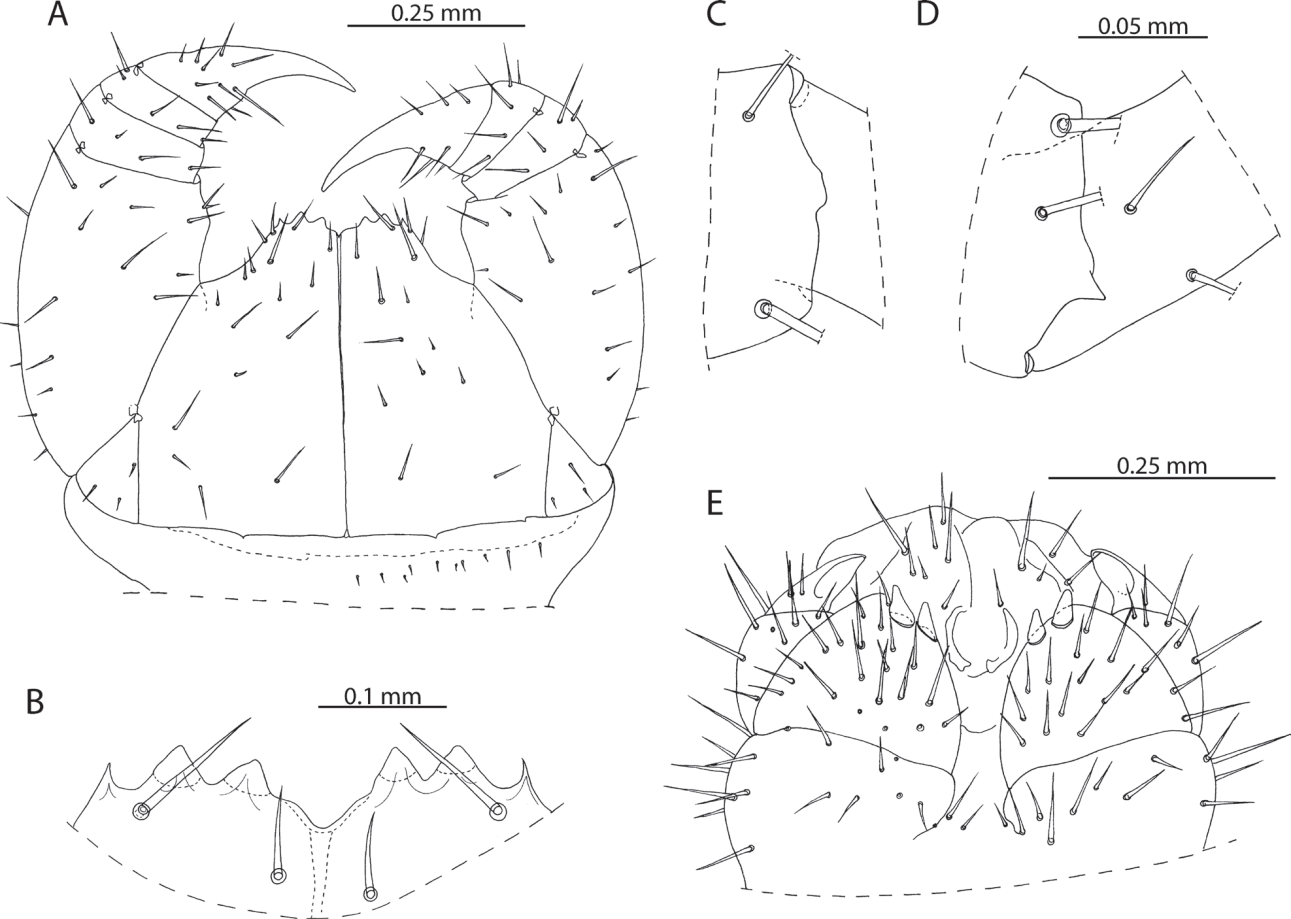
*Lamyctestristani* (Pocock, 1893) **A, B, D** NHMUK015991452 **A** forcipular segment, ventral view **B** anterior margin of forcipular coxosternite, ventral view **C** NHMUK015991458, distal end of tibia of leg pair 12, lateral view **D** distal end of tibia of leg pair 12, lateral view **E** NHMUK015991456, Female gonopods, ventral view.

The 38 specimens from the Aldabra Atoll (from Cinq Cases, Malabar, and Picard) for which sex can reliably be determined are all females, such that the population is parthenogenetic. They are likely conspecific with *L.tristani* as described from the Chagos Archipelago (Popovici et al. 2024), sharing the distinctly reduced spinous process on the tibia of leg pair 12, often taking the form of a rounded bump (Fig. 1C). The stability of this character is however uncertain as specimens from localities in the Aldabra Atoll exhibit conspicuous variation in the shape of the process (Fig. 1D, Table 1). Additional similarities between these two populations include the morphology of the forcipular coxosternite (Fig. 1A, B), the number of antennal articles, and the number and shape of the spurs on the female gonopods (Fig. 1E). Variability in the characters of antennal article number, number, and distribution of coxal pores and shape of the distal spinous process on the tibia of leg pair 12 are listed in Table 1 and compared with putatively conspecific individuals from the Chagos Archipelago, excluding anamorphic stages.

**Table 1.** Morphological variability of putative *L.tristani* specimens from Western Indian Ocean localities. Asterisk indicating regenerated appendage.

Locality	Body length (mm)	Antennal article number	Coxal pore formula (legs 12/12 – 13/13 – 14/14 – 15/15)	Shape of tibial spinous process on leg pair 12
Cinq Cases (Aldabra)	3.5	20	1/1 – 1/1 – 1/1 – 1/1	Rounded bump
Cinq Cases (Aldabra)	4	20	1/1 – 1/1 – 1/1 – 1/1	Rounded bump
Cinq Cases (Aldabra)	5	17	2/2 – 2/2 – 3/3 – 2/2	Acuminate, minute
Cinq Cases (Aldabra)	5	22	3/3 – 3/3 – 3/3 – 2/2	Acuminate, minute
Cinq Cases (Aldabra)	5	-	2/2 – 3/3 – 3/3 – 2/2	-
Cinq Cases (Aldabra)	5	22	2/2 – 3/3 – 3/3 – 2/2	Rounded bump
Cinq Cases (Aldabra)	6	23	3/3 – 3/3 – 3/3 – 3/3	Acuminate, minute
Malabar (Aldabra)	5.5	24	2/2 – 2/2 – 3/3 – 2/2	-
Malabar (Aldabra)	5.5	24	2/2 – 2/2 – 3/3 – 2/2	Rounded bump
Malabar (Aldabra)	6	(23)24	2/2 – 2/2 – 2/3 – 2/2	Rounded bump
Malabar (Aldabra)	6	15(20)	3/3 – 3/3 – 3/3 – 3/3	Asymmetrical, rounded bump on left tibia, acuminate spur on right tibia
Malabar (Aldabra)	6	24	3/3 – 3/3 – 3/3 – 3/3	-
Picard (Aldabra)	3.5	19	1/1 – 1/1 – 1/1 – 1/1	Rounded bump
Picard (Aldabra)	3.5	20	1/1 – 1/1 – 1/1 – 1/1	Rounded bump
Picard (Aldabra)	3.5	20	1/1 – 1/1 – 1/1 – 1/1	Rounded bump
Picard (Aldabra)	4	20	1/1 – 1/1 – 1/1 – 1/1	Rounded bump
Picard (Aldabra)	4	20	1/1 – 1/1 – 1/1 – 1/1	Rounded bump
Picard (Aldabra)	4	20	1/1 – 1/1 – 1/1 – 1/1	Rounded bump
Picard (Aldabra)	4	20	1/1 – 1/1 – 1/1 – 1/1	Rounded bump
Picard (Aldabra)	4	21	2/2 – 2/2 – 2/2 – 1/1	Rounded bump
Picard (Aldabra)	4	21	2/2 – 2/2 – 2/2 – 1/1	Rounded bump
Picard (Aldabra)	4	20	1/1 – 1/1 – 1/1 – 1/1	-
Picard (Aldabra)	4.5	24	2/2 – 2/2 – 3/3 – 2/2	Acuminate, minute
Picard (Aldabra)	4.5	(22)23	2/2 – 2/2 – 3/3 – 2/2	Rounded bump
Picard (Aldabra)	4.5	21	1/1 – 2/2 – 2/2 – 2/2	-
Picard (Aldabra)	5	(24)25	2/2 – 2/2 – 2/2 – 2/2	Rounded bump
Picard (Aldabra)	5	(22)23	3/3 – 3/3 – 3/3 – 2/2	Acuminate, minute
Picard (Aldabra)	5	15*	3/3 – 3/3 – 3/3 – 2/2	Acuminate, minute
Picard (Aldabra)	5	24	2/2 – 3/3 – 3/3 – 2/2	Acuminate, minute
Picard (Aldabra)	5	(21)24	2/2 – 3/3 – 3/3 – 2/2	Acuminate, minute
Picard (Aldabra)	5	-	2/2 – 3/3 – 3/3 – 2/2	Acuminate, minute
Picard (Aldabra)	5	24	2/2 – 3/3 – 3/3 – 2/2	Acuminate, minute
Picard (Aldabra)	5	20	2/2 – 2/2 – 3/3 – 2/2	Rounded bump
Picard (Aldabra)	5.5	24	3/3 – 3/3 – 3/3 – 3/3	Acuminate, minute
Picard (Aldabra)	6	24	3/3 – 3/3 – 3/3 – 3/3	Acuminate, reduced in size
Picard (Aldabra)	7	(24)25	3/3 – 3/3 – 3/3 – 3/3	Acuminate, reduced in size
Diego Garcia (Chagos)	6	27	3/3 – 3/3 – 3/3 – 3/3	Rounded bump
Diego Garcia (Chagos)	6.2	(18)20	3/3 – 3/3 – 3/3 – 3/3	Rounded bump
Diego Garcia (Chagos)	6.5	(21)24	3/3 – 3/3 – 3/4 – 4/4	Rounded bump

###### ﻿Order Scolopendromorpha

**Family Cryptopidae**

**Genus *Cryptops* Leach, 1815**

#### ﻿ Cryptops cf. japonicus

Taxon classificationAnimaliaScolopendromorphaCryptopidae

Takakuwa, 1934

DC1DCBCA-CB15-5101-A237-0EB887D8E379

Fig. 2

##### Examined material.

35 specimens: NHMUK015991425, 4 specimens, Black Path, Picard, Summer 1975, leg. V. W. Spaull; NHMUK015991429, 1 specimen, Aldabra, 10.11.1973; NHMUK015991430, 6 specimens, Point Hodoul, Grande Terre, 22.03.1974; NHMUK015991431, 2 specimens, Ile. Malabar, 08.06.1974; NHMUK015991432, 18 specimens, *Casuarina* and *Sideroxylon* litter, Anse Cedres, 12.02.1974; NHMUK015991433, 4 specimens, Esprit, 14.12.1974.

##### Remarks.

**Figure 2.**
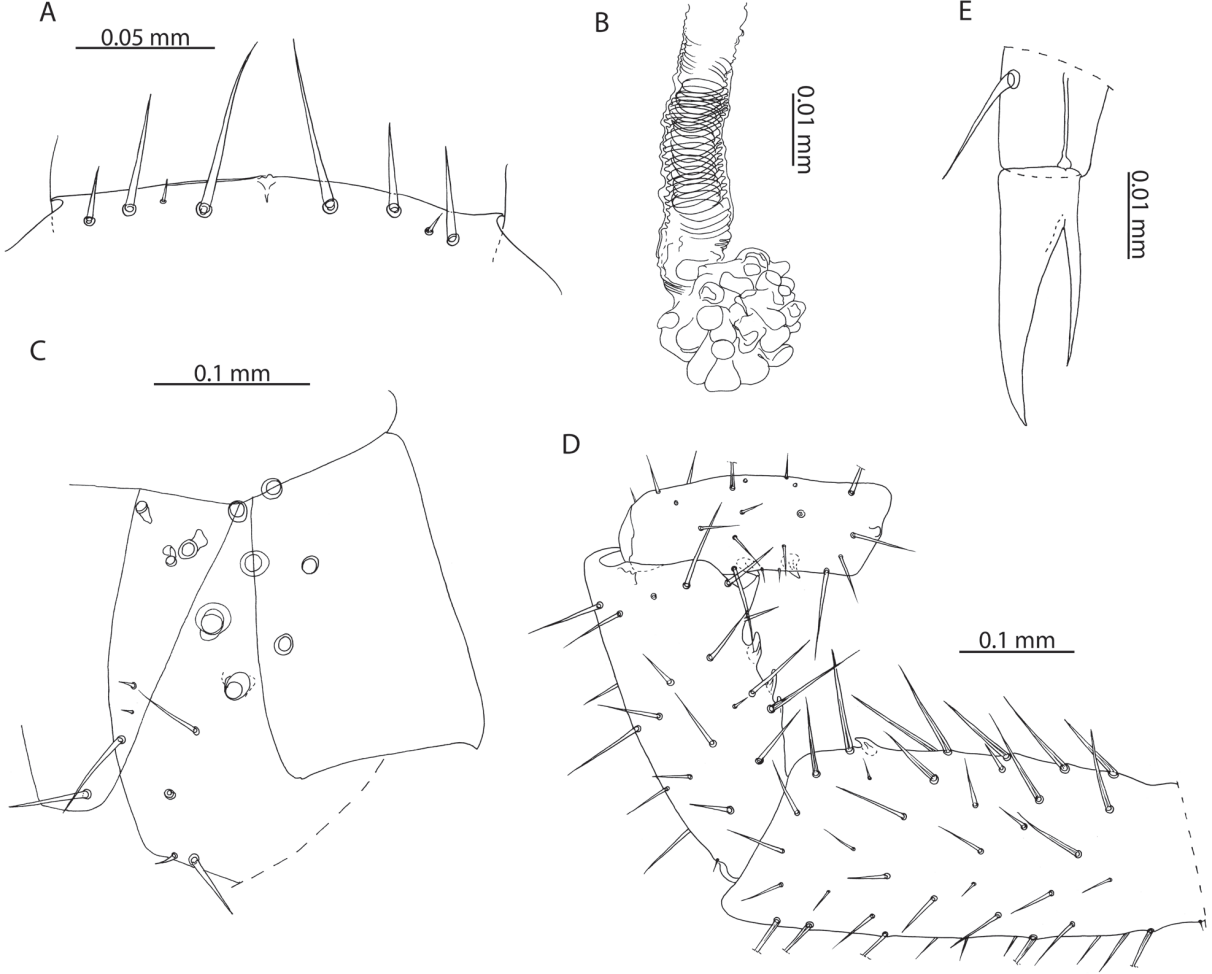
Cryptopscf.japonicus Takakuwa, 1934 **A, B, D** NHMUK015991433 **A** anterior margin of forcipular coxosternite, ventral view **B** Calyx of venom gland, lateral view **C** NHMUK015991430, coxopleuron of ultimate leg-bearing segment, lateral view **D** femur, tibia, and tarsus 1 of ultimate leg telopodite, lateral view **E**. NHMUK015991429, pretarsus of leg pair 8, lateral view.

Cryptopscf.japonicus collected in Aldabra is an unexpected occurrence for a species otherwise restricted to localities in southern Japan, the Korean peninsula, Manchuria (Takakuwa 1936) and Taiwan (Chao 2008). Despite the description of the morphologically similar *C.doriae* Pocock, 1891 from nearby localities in the Seychelles (Lewis 2007a), consistent diagnostic morphological characters separate these two populations. Most prominently, specimens from Aldabra only have one pretarsal accessory spur on legs 1–20, > 1/2 the length of the pretarsus (Fig. 2E), in contrast to *C.doriae* from the Seychelles which has been described with two generally subequal, conspicuous, accessory spurs that are much shorter relative to the pretarsus, on the same leg pair range (Lewis 2007a). The examined specimens agree with the description provided by Chao (2008) based on specimens collected in Taiwan, in which he notes the low number of coxopleural pores (9) in the immature (“larva”) stages, which overlaps with the condition of Aldabra specimens (Fig. 2C), the presence of 4 setae along the anterior margin of the forcipular coxosternite (Fig. 2A), and the ovoid shape of the calyx of the venom gland (Fig. 2B).

All specimens from Aldabra Atoll range from 3–10 mm and exhibit several traits characteristic of juvenile specimens including reduced number of pores on the coxopleuron, indistinct paramedian sutures on tergites, and a reduced number of tibial and tarsal saw teeth (Fig. 2D). Following clearing, no spermatozoa or oocytes could be observed in the posterior trunk of specimens. Without additional sampling to confirm the condition of adult specimens it is not possible to comment on the presence or absence of sexually mature adults in the present sample. Introduced populations of *C.doriae* have been described bearing similar neotenic characteristics (reduced body size, number of coxal pores, number of saw teeth), even in sexually mature adults (Lewis 2007b), potentially explaining the morphology of the Aldabra specimens in light of possible introduction to the atoll. Nevertheless, in the absence of additional material and molecular data, our assignment to *Cryptopsjaponicus* is only tentative. The status and relationships of different populations identified as *C.doriae* and related taxa remains to be clarified.

#### ﻿ Cryptops mauritianus

Taxon classificationAnimaliaScolopendromorphaCryptopidae

Verhoeff, 1939

AAAF8A3F-EADF-5FE8-AA0F-4EB1D1E3E2D4

Fig. 3

##### Examined material.

NHMUK015991434, 1 specimen, *Sideroxylon* litter, Anses Coco & Porche, Aldabra, 03.12.1974, V. W. Spaull. leg.

##### Remarks.

**Figure 3.**
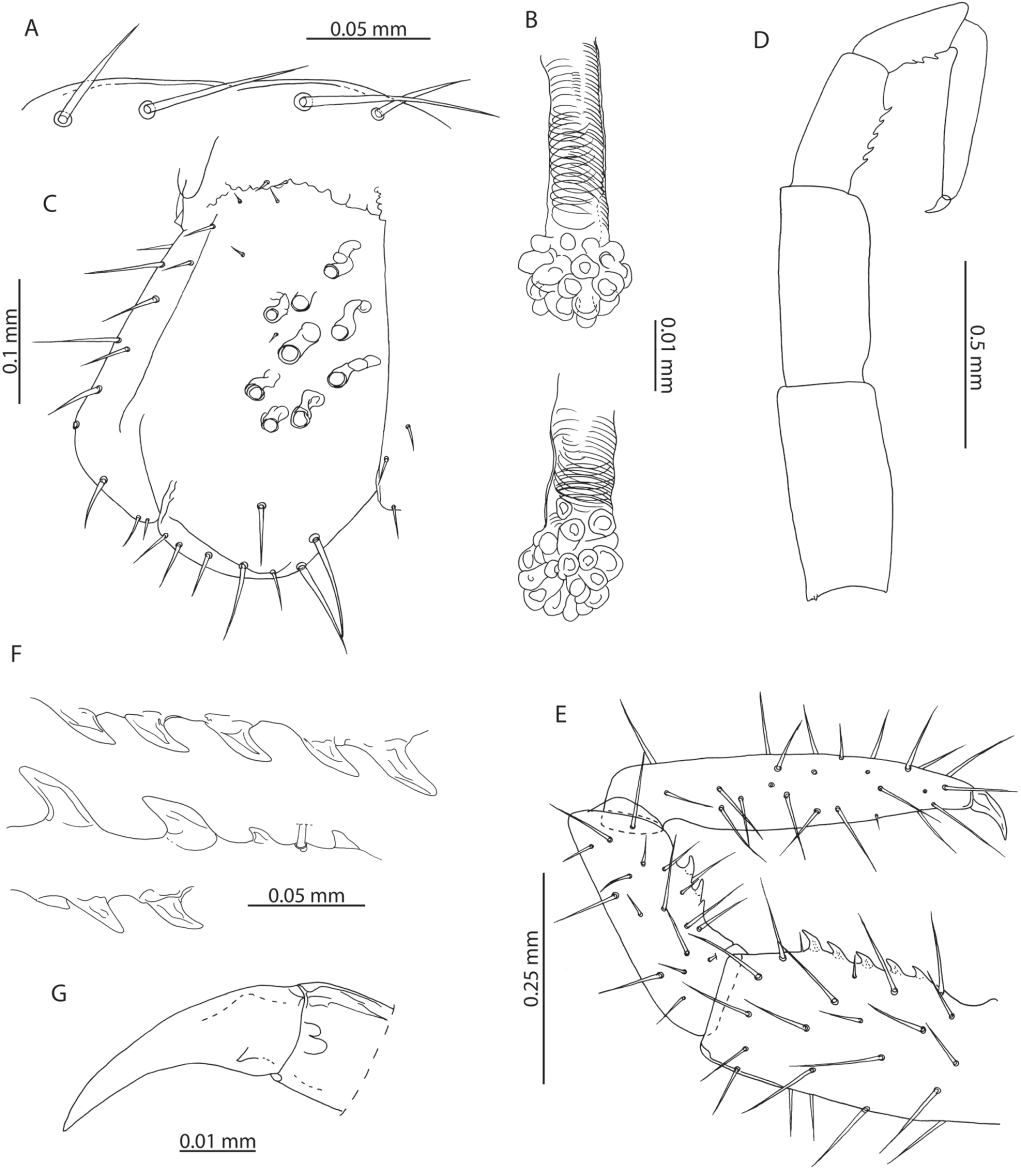
*Cryptopsmauritianus* Verhoeff, 1939. NHMUK015991434 **A** anterior margin of forcipular coxosternite, ventral view **B** calyx of venom gland. Left forcipule (top), right forcipule (bottom) **C** coxopleuron of ultimate leg-bearing segment, lateral view **D** Ultimate leg telopodite, lateral view **E** tibia and tarsal articles of ultimate leg, lateral view **F** tibial comb (top), tarsal comb of left ultimate leg (middle), tarsal comb of right ultimate leg (bottom) **G** pretarsus of leg pair 18, lateral view.

*Cryptopsmauritianus* has been described by Verhoeff (1939) from Mauritius. Subsequent taxonomic revision of Mauritian *Cryptops* species (Lewis 2002) completed the summary original description and provided additional illustrations of material from near the type locality. In the singular Aldabra specimen examined, the presence of minute accessory spurs on the pretarsus of leg pairs 1–20 (Fig. 3G) clearly delimits it from all other *Cryptops* species known from localities in the Western Indian Ocean (Lewis 2011). Subsequent re-description by Lewis (2002) provided data on an immature (“adolescens”) specimen which matches the condition of the material presently examined (body length 8 mm), allowing us to confidently refer it to *C.mauritianus*.

#### ﻿ Cryptops nigropictus

Taxon classificationAnimaliaScolopendromorphaCryptopidae

Takakuwa, 1936

ADBE6217-5EF0-5AEA-9D94-A0DE96CB9C55

Fig. 4

##### Examined material.

12 specimens: NHMUK015991424, 3 specimens, Black Path, Picard, Aldabra, Summer 1975; NHMUK015991426, 3 specimens, *Calophyllum* litter, Takamaka, Grande Terre, 14.01.1975; NHMUK015991427, 2 specimens, *Sideroxylon* litter, Au Parc, Aldabra, 14.02.1975. V. W. Spaull leg; NHMUK015991428, 1 specimen, Picard, Aldabra, 28.02.1975; NHMUK015991445, 1 specimen, *Sideroxylon* litter, Ile. Michel, Aldabra, 28.03.1975; NHMUK015991446, 2 specimens, *Casuarina* litter, Picard, Aldabra, 08.05.1974.

##### Remarks.

**Figure 4.**
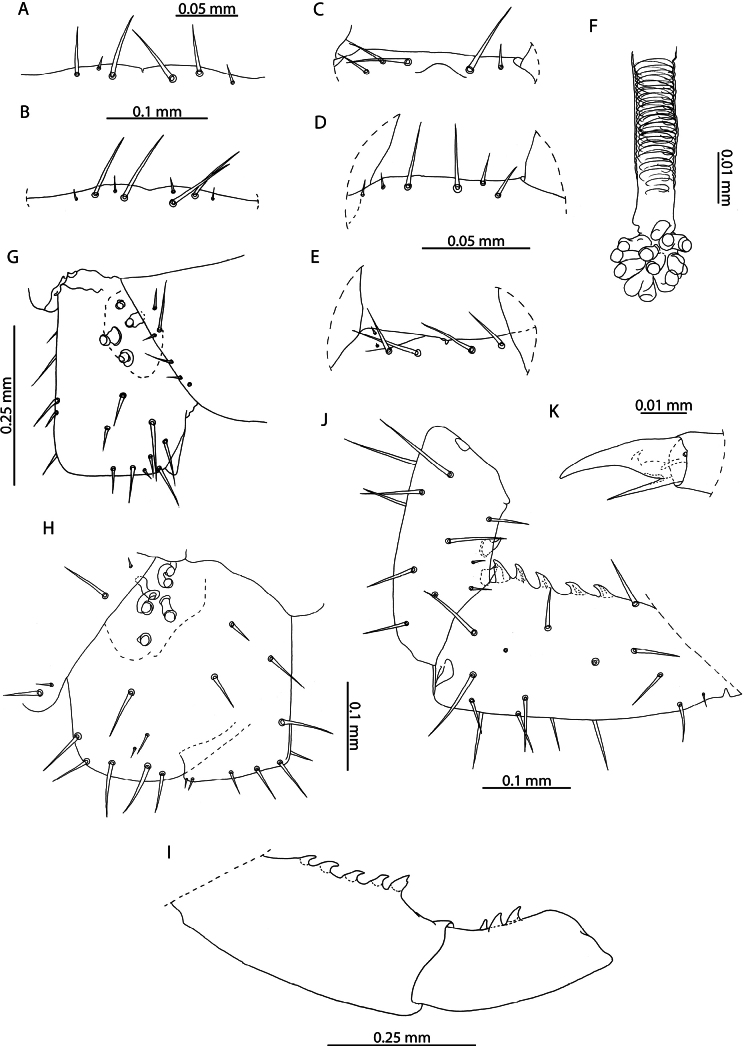
*Cryptopsnigropictus* Takakuwa, 1936 **A, F, K, I, B** NHMUK015991426 **H, J** NHMUK015991446 **G** NHMUK015991424 **C** “*Cryptopsdaszaki*” holotype, Île aux Aigrettes, 19.10.1995 **D, E** paratype, Île aux Aigrettes, 18.10.1995 **A, B, C, D, E** anterior margin of forcipular coxosternite, ventral view **F** calyx of venom gland, lateral view **G, H** coxopleuron of ultimate leg-bearing segment, lateral view **I, J** tibia and tarsus of ultimate leg telopodite, lateral view **K** pretarsus of leg pair 8, lateral view.

Taxonomic revision of *Cryptops* species belonging to the “*hortensis* group” identified *C.decoratus* Lawrence, 1960, *C.melanotypus* Chamberlin, 1941, and *C.nigropictus* Takakuwa, 1936 as a potential species complex, raising doubts on the taxonomic validity of many morphologically similar species (Lewis 2011). A reliable diagnostic trait mentioned by Lewis (2011) is the presence of a single long pretarsal accessory spur in *C.nigropictus* (Fig. 4K), differentiating it from *C.decoratus* and *C.melanotypus* which bear two small pretarsal accessory spurs on leg pairs 1–20. The Aldabra specimens key out to *C.nigropictus* in the key provided within the same article and agree with its revised description. Although originally described from East Asia, various nominal taxa recorded from East Africa and islands of the Indian Ocean (including Mauritius and Rodrigues) have been placed in synonymy with *C.nigropictus* (Lewis 2011).

*Cryptopsdaszaki* Lewis, 2002 was described from several localities in Mauritius. Lewis (2002) remarked on the small body size (4.5–7.5 mm) of sexually mature specimens and their apparent juvenile characteristics (low number of coxal pores, reduced setation). The specimens collected from Aldabra with a similar body length to specimens assigned by Lewis to *C.daszaki* overlap in morphology with respect to the usual lack of subcuticular dark pigment, the number of setae on the anterior margin of the forcipular coxosternite (2 or 3 on each side in *C.daszaki*, 3 or 4 on each side in *C.nigropictus*; Fig. 4C versus Fig. 4A, B, D, respectively), the relative length of the single pretarsal accessory spur on legs 1–20 (> 1/2 the length of the pretarsus) and the number of coxal pores (5 or 6 in *C.daszaki*, 4–9 in *C.nigropictus*) but show greater variation than *C.daszaki* in the number of saw teeth on the tibia (3–5) and tarsus (2 or 3). The number of tibial and tarsal saw teeth is known to be intraspecifically variable in *Cryptops* (Iorio and Geoffroy 2003; Lewis 2009) and scales allometrically with body size, as exemplified by the largest *C.nigropictus* specimen in our sample (12 mm, NHMUK015991426; Fig. 4I).

The only other diagnostic trait given by Lewis (2011) separating *C.daszaki* and *C.nigropictus* is the position of the setae near the anterior margin of the forcipular coxosternite, being described as “marginal” in *C.daszaki* and “submarginal” in *C.nigropictus*. Re-examination of the type specimens of *C.daszaki* revealed that these setae occupy a submarginal position (Fig. 4C), which may be misinterpreted as marginal depending on the orientation of the specimens. As both of the two putative diagnostic traits separating *C.daszaki* and *C.nigropictus* are fully encompassed by intraspecific variation within *C.nigropictus* and the latter species has been recorded from nearby localities (Lewis 2002), we consider *C.daszaki* a likely junior subjective synonym of *C.nigropictus*, from which it cannot be reliably distinguished by morphology alone. Similarly, the presence of subcuticular dark pigment cannot be reliably used to separate putative *Cryptops* species in Western Indian Ocean and Eastern African localities. This character is variable within our sample, with all specimens with the exception of NHMUK015991428 lacking dark subcuticular pigment. Dark pigmentation is also variable in the type specimens of *Cryptopsniloticus* Lewis, 1967 (present in male allotype but absent in female holotype and in juvenile paratype), a species previously recorded from the Western Indian Ocean (Lewis 2002) but subsequently synonymised with *C.nigropictus* (Lewis 2011).

###### ﻿Family Scolopendridae

**Genus *Scolopendra* Linnaeus, 1758**

#### ﻿ Scolopendra morsitans

Taxon classificationAnimaliaScolopendromorphaScolopendridae

Linnaeus, 1758

519BCC2A-8AB5-583C-B0F4-24C9519743F9

##### Examined material.

52 specimens: NHMUK015991435, 33 juveniles; *Casuarina* litter, Picard, Aldabra, 18.04.1974, leg. V. W. Spaull; NHMUK015991436, 1 specimen, *Casuarina*, Picard, Aldabra, 04.02.1974; NHMUK015991437, 1 specimen, *Ochna* soil, Picard, Aldabra, 15.02.1974; NHMUK015991438; 2 specimens, mixed scrub, Picard, 24.12.1974; NHMUK015991439, 1 specimen, *Thespesia* litter, Cinq Cases, Aldabra, 15.11.1973; NHMUK015991441, 5 specimens, Black Path, Picard, 1975; NHMUK015991442, 1 specimen, Picard, 03.12.1973; NHMUK015991443, 1 specimen, Picard, 18.01.1974; NHMUK015991440, 4 specimens, Picard, 18.11.1974; NHMUK015991444, 1 specimen, Pitfall trap 5, 08.12.1974; NHMUK015991447, 1 specimen, South Island, Aldabra, 13–20.03.1968, leg. B. Cogan & A. Hutson; NHMUK015991448, 1 specimen, Ile. Michel, 02.1968, leg. B. Cogan & A. Hutson.

##### Remarks.

Specimens of *S.morsitans* Linnaeus, 1758 collected from mainland Africa, originally identified as *Scolopendraamazonica* Bücherl, 1946 overlap with the specimens from the Aldabra Atoll in several characters (Table 2) (Lewis 1966, 1967, 1968, 1969), but generally have a greater number of glabrous basal antennal articles, a character in which specimens from the Aldabra Atoll more closely match Indian specimens of *S.morsitans* previously assigned to *S.amazonica* (Jangi 1955, 1959). In the absence of newly collected material from which molecular data can be collected to evaluate possible interspecific delimitation between different population of *S.morsitans*, we assign material collected in the Aldabra Atoll to *S.morsitans*, following the conclusions on interspecific variation within this taxon reached by Würmli (1975). Subsequent phylogenetic analyses of molecular data for *S.morsitans* identified multiple lineages within this taxon (Joshi and Karanth 2011; Siriwut et al. 2016), potentially indicating the existence of a cryptic species complex as suggested by recent taxonomic review (Lewis 2010a). Until the global taxonomy of *S.morsitans* is interrogated using molecular data to establish if this is the case, we classify the examined material as *S.morsitans*.

**Table 2.** Morphological variability in *Scolopendramorsitans* populations obtained from literature data (Lewis 1966, 1967, 1969) and examined specimens.

Character	*Scolopendramorsitans* (India)	*Scolopendraamazonicasensu* Jangi (= *Scolopendramorsitans*) (India)	*Scolopendraamazonicasensu* Lewis (= *Scolopendramorsitans*) (Africa)	*Scolopendramorsitans* (Aldabra)
Subadult and adult body length (mm)	18–113	15–65	13–100	13–78
Number of antennal articles	20	19	18–21	(17)18–19
Number of glabrous antennal articles	Can be > 6	≤ 6	5–7	(3.5)4
Complete paramedian sutures begin	Mostly T3	Mostly T2	TT2–4	T3
Lateral margination	May begin more anteriorly	Generally last 5 tergites	Last 2–15 tergites	Last 4–10 tergites
Coxopleural process spines	5	4	2–6	(3)4–5
Lateral coxopleural spine	Present	Present or absent	Present or absent	Present
Leg 20 tarsal spur	Present	Absent	Absent	Absent

Additionally, specimens were compared to the original description of the morphologically similar and geographically proximate *Scolopendraantananarivoensis* Kronmüller, 2010. Material from the Aldabra Atoll did not exhibit the characters given as diagnostic for *S.antananarivoensis*, lacking a longitudinal median depression on sternite 21 and not having a distinctly more elongate coxopleural process (Kronmüller 2010).

###### ﻿Order Geophilomorpha

**Family Geophilidae**

**Genus Ribautia Brölemann, 1909**

#### ﻿ Ribautia cf. paucipes

Taxon classificationAnimaliaGeophilomorphaGeophilidae

Attems, 1952

8558B297-0A00-59EF-8D8D-9517C5F0B6D5

Fig. 5

##### Examined material.

NHMUK015991467, 1 juvenile, *Casuarina* litter, Picard, Aldabra, 10.12.1974, leg. V. W. Spaull.

##### Remarks.

**Figure 5.**
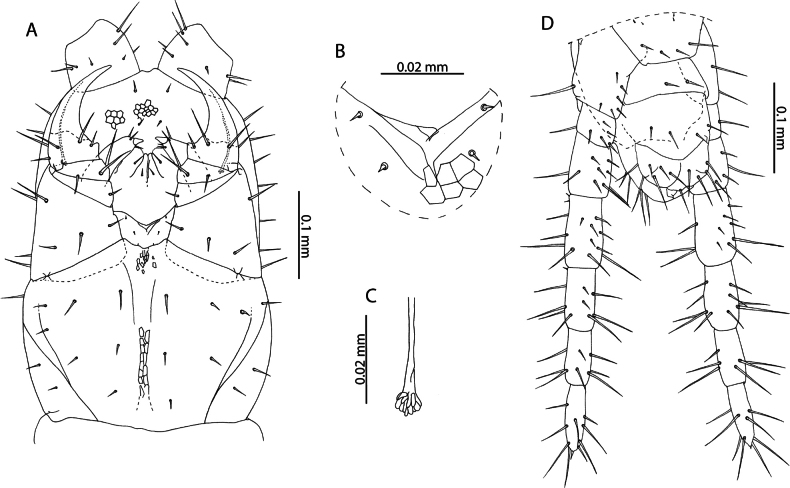
Ribautiacf.paucipes Attems, 1952. NHMUK015991467 **A** head and forcipular segment, ventral view **B** anterior margin of second maxillary coxosternite, ventral view **C** calyx of venom gland, lateral view **D** ultimate leg-bearing and postpedal segments, ventral view.

The sexually immature specimen found in the present sample displays all diagnostic characters that support its assignment to *Ribautia*, comprising an elongate cephalic shield, lack of lappets on the first maxillae, the two halves of the second maxillary coxosternite being united by a sclerotised isthmus and the pleural sutures of the forcipular coxosternite being parallel to its lateral edge distally. A potentially novel ontogenetic observation is the incomplete separation of the two halves of the second maxillary coxosternite by an isthmus (Fig. 5B), as is a characteristic of *Ribautia*. Taking into account the very small size (length 7 mm) and sexual immaturity of the specimen, this may be a character state that becomes more conspicuous in older individuals.

Beside the very low number of leg-bearing segments (37), which is shared with R.cf.paucipes reported from the Seychelles (Bonato and Minelli 2010), the developmental stage of the present specimen does not allow for satisfactory evaluation of potential morphological differences between *Ribautia* specimens described from Western Indian Ocean localities and type material of *Ribautiapaucipes* described from the environs of Lake Kivu in Central Africa. Notably, the Aldabra specimen lacks evident denticles on the anterior margin of the forcipular coxosternite and the interior margin of the forcipular trochanteroprefemur, although these are clearly illustrated in the original description of *R.paucipes* (Attems 1952: fig. 203). The specimen also lacks conspicuous coxal organs or coxal pores, a character state not noted in R.cf.paucipes recorded from the Seychelles. Ontogenetic variation in the number of coxal pores has been well-documented for other geophilid centipedes (Horneland and Meidell 2009; Gregory and Barber 2010; Brena 2014; Stojanović et al. 2020), increasing with body size at each postembryonic stage, and being absent in comparably sized adolescens stages of some species (Gregory and Barber 2010). Additionally, the second maxillary pretarsus of the present specimen is markedly shorter and less acuminate than illustrated for *R.paucipes* from continental Africa. As the ontogenetic variation of the morphology of the second maxillae in *Ribautia* is not presently known and in other characters the specimen strongly resembles individuals described from the Seychelles, we maintain its presently assigned identity.

###### ﻿Genus *Tuoba* Chamberlin, 1920

***Hovanyx* Lawrence, 1960, syn. nov.**

#### ﻿ Mixophilus

Taxon classificationAnimaliaGeophilomorphaGeophilidae

Silvestri, 1929 syn. nov.

5BC7E5A9-6EDC-5799-B2F0-9C1E633A4CE6

##### Remarks.

The monotypic genus *Mixophilus* was erected by Silvestri (1929) to place a new species of geophilomorph sampled from riparian sites in Madras (Chennai), Southern India. The original description of *Mixophilusindicus* includes illustrations of the head, forcipular apparatus and ultimate leg-bearing segment as well as detailed ecological observations concerning its preferred microhabitats in the type locality. Subsequent physiological investigations revealed a modified tracheal system, comprising possible adaptations to immersion for long periods of time (Rajulu 1970). An affinity to *Henia* and *Chaetechelyne* (Dignathodontidae) was suggested, however, within the same section Silvestri (1929) pointed to differences between *Mixophilus* and both of these genera in “the structure of the labrum” (tripartite in *M.indicus* but unipartite in Dignathodontidae), “the distribution of sternal pores” (transverse band in *M.indicus*, medial sub-circular/elliptical field in Dignathodontidae), in the last leg-bearing segment (telopodite composed of 7 articles in *M.indicus* but 6 in *Henia*), and in the elongation of the forcipular pretergite (longer in *M.indicus* than in *Chaetechelyne*). The structure of the labrum described and illustrated for *M.indicus*, comprising three conspicuous pieces, suggests a close affinity to other members of Geophilidae s. str. An estuarine habitat preference and multiple morphological characters show a near complete overlap between the diagnoses of *Tuoba* and *Mixophilus* (Table 3). Complete chitin lines in *M.indicus* may indicate intraspecific or interspecific variability which has been recorded in *Tuoba* (Jones 1998) or artifacts of examination under light microscopy. In light of this re-evaluation of its original description, we propose reassignment of *M.indicus* to *Tuoba* considering the available data on its morphology, with *Tuobaindica* comb. nov. as the valid name for specimens on which its original description was based. Consequently, we propose that *Mixophilus* is the junior subjective synonym of *Tuoba* syn. nov. Similarities between *Tuobaindica* comb. nov. and *T.sydneyensis* exist in elongation of the ultimate legs and reduction of the second maxillary pretarsus, however these characters are shared by several species of *Tuoba*. The incomplete original description provided by Silvestri does not allow for definitive assignment of *Tuobaindica* comb. nov. to another species of *Tuoba* until the type material can be adequately re-described.

**Table 3.** Taxonomically informative invariant and variable morphological characters for the genera *Hovanyx*, *Mixophilus*, and *Tuoba* based on literature data. Characters in boldface represent putative morphological differences.

Character	*Tuoba* Chamberlin, 1920	*Hovanyx* Lawrence, 1960	*Mixophilus* Silvestri, 1929
Head shape	Subquadrate	**Longer than wide**	Subquadrate
Setation of clypeus	Three pairs of setae medially, flanked by a group of 2–4 setae on each side	10–11 setae	Four pairs of setae medially, flanked by a group of 3 or 4 setae on each side
Medial piece of labrum. Orientation of tubercles	Anteriorly recurved (variable)	Anteriorly recurved	Anteriorly recurved
Side pieces of labrum	With variable number of tubercles or plumose setae	-	Without tubercles or plumose setae
Maxillae I lappets	Absent. External corners with spiniform cuticular projections	Absent. External corners with spiniform cuticular projections	Absent
Maxillae II pretarsus	Simple, claw-shaped. Variably reduced in size	**Simple, claw-shaped**	Simple, claw-shaped. Reduced in size
Forcipular coxosternite chitin lines	Complete or nearly complete	Vanishing before reaching the condyles	Complete
Denticle at the base of the tarsungulum	Present	Present	Present
Carpophagus structures	**Present**	-	-
Metasternal pore field shape	Transverse band	Transverse band. Divides on LBS XI–XII	Transverse band (medially constricted)
Pretarsus of walking legs	Distinctly elongate	Distinctly elongate	Distinctly elongate
LLBS metasternite shape	Wider than long, trapezoidal	Wider than long, trapezoidal	Wider than long, trapezoidal
Coxal organs	Multiple opening in single pit	**Absent**	Multiple opening in single pit
LLBS pretarsus	Simple, claw-shaped	Simple, claw-shaped	Simple, claw-shaped

Similarly, the genus *Hovanyx* was erected for the species *Hovanyxwaterloti*, described in Lawrence’s (1960) catalogue of Malagasy centipedes. An affinity to Dignathodontidae was again proposed based on similarities in the structure of the labrum, originally described for *H.waterloti* as composed of a single piece and bearing a small number of rudimentary, anteriorly oriented tubercles (“Labre […] à dents pas très distinctes, quatre courtes dents triangulaires dirigées vers l’avant, de chaque côté.”). The diagnosis of *Hovanyx* singled out the absence of coxal organs (“[…] diffère de tous les autres membres de la sous-famille des Dignathodontinæ par l’absence de pores aux pattes terminales.”) as the main distinguishing trait separating it from all other dignathodontid genera. However, both the original description and accompanying illustrations suggest a closer affinity to Geophilidae s. str., as multiple other morphological characters (shape of head, forcipular coxosternite, metasternal pore fields) are characteristic of the Geophilidae rather than the Dignathodontidae, and subsequent taxonomic revision of both families placed *Hovanyx* under Geophilidae (Bonato 2011). Furthermore, the incomplete original description overlaps almost entirely with that of *T.sydneyensis*, a wide-ranging geophilid encountered in littoral sites in the Seychelles (Bonato and Minelli 2010) and the Aldabra Atoll (present records) close to the type locality of *H.waterloti*. Shared characters include range of leg bearing segment number (41–43), morphology of the labrum, condition of chitin lines on the forcipular coxosternite (incomplete) (Table 3). The only putative differences between *H.waterloti* and *T.sydneyensis* are the unipartite labrum and absence of coxal organs in the former (Table 3). These may however be unreliable characters because of inadequate documentation, as published illustrations of *Tuoba* specimens and the material here illustrated show great variability in the orientation and shape of the labral pieces, which depending on the degree of sclerotization seen in the specimen and shape and number of denticles on the side pieces, may resemble the labrum of Dignathodontidae under light microscopy. The absence of coxal organs has not been previously reported in any species of *Tuoba*, however this character shows extensive ontogenetic plasticity within Geophilidae (Horneland and Meidell 2009; Gregory and Barber 2010; Tuf and Dányi 2015; Peretti and Bonato 2016; Stojanović et al. 2020) and the small size of the holotype and only known specimen as well as variations in clearing and position of the coxopleuron may render the pit inconspicuous.

#### ﻿ Tuoba sydneyensis

Taxon classificationAnimaliaGeophilomorphaGeophilidae

(Pocock, 1891a)

4FD2EFF6-F9BE-5603-832C-3466D1B6B45B

**Figure 6.**
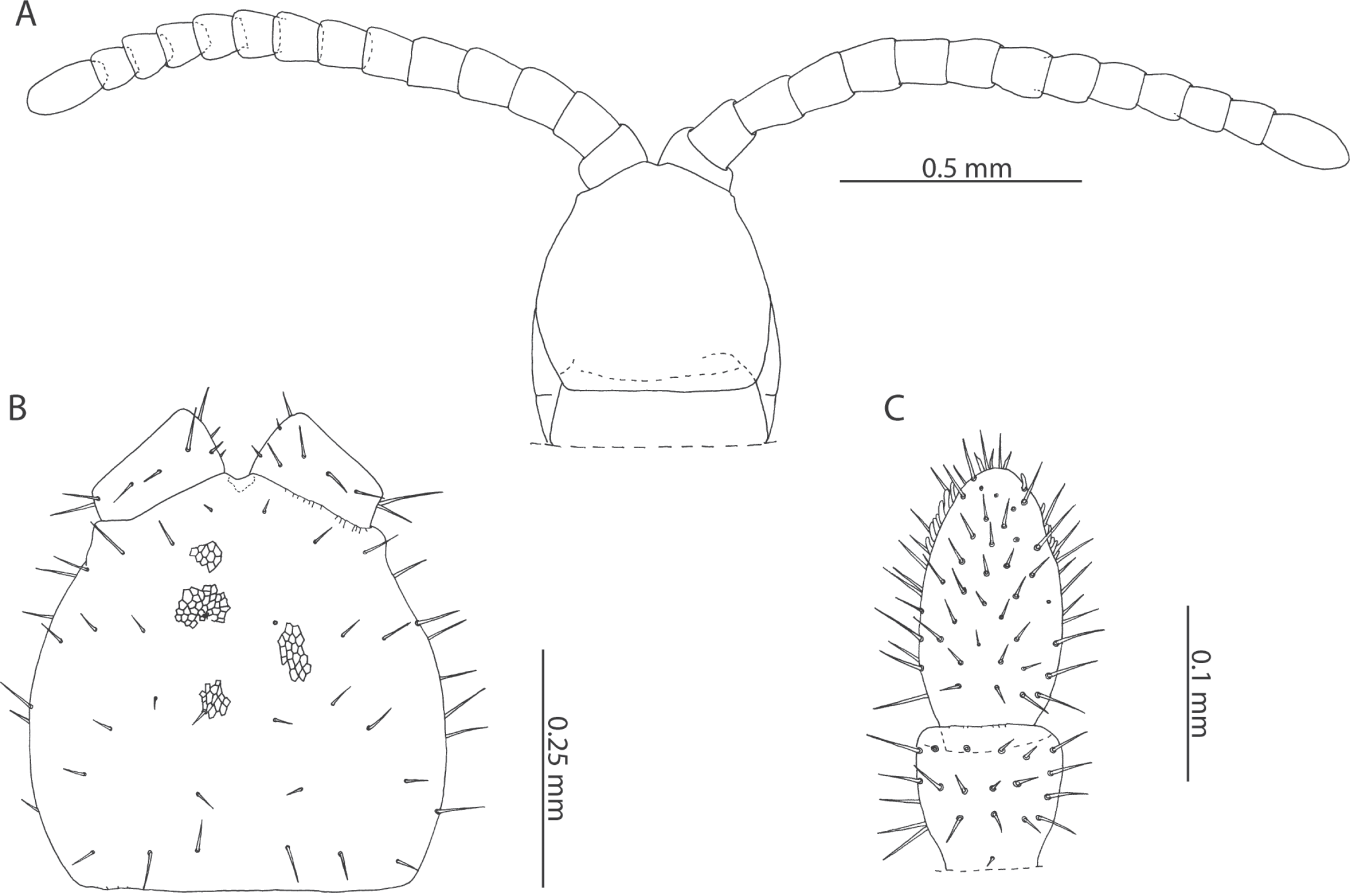
*Tuobasydneyensis* (Pocock, 1891a). NHMUK015991484 **A** head and antennae, dorsal view **B** cephalic shield, dorsal view **C** antennal article XIV, dorsal view.

**Figure 7.**
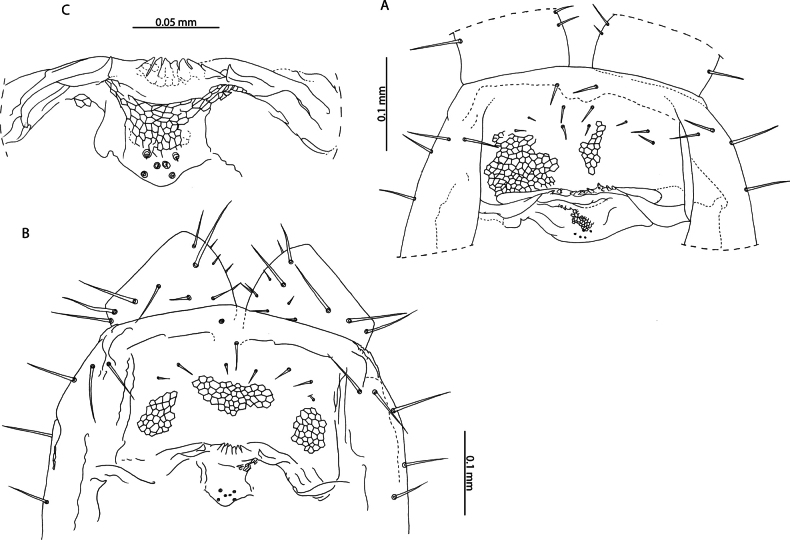
*Tuobasydneyensis* (Pocock, 1891a) **B, C** NHMUK015991478 **A** NHMUK015991484 **B** clypeus and labrum, ventral view **C** labrum, ventral view.

**Figure 8.**
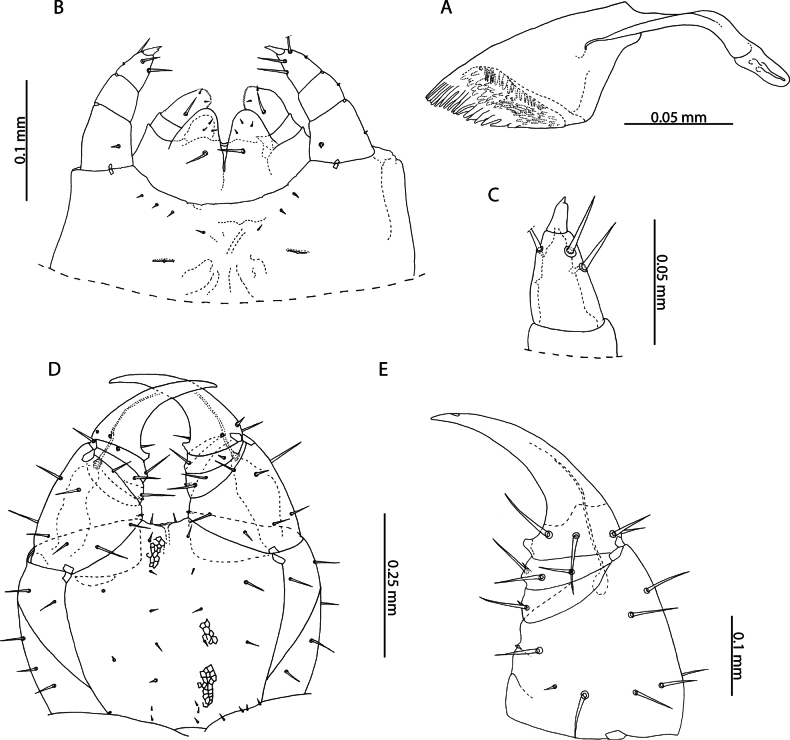
*Tuobasydneyensis* (Pocock, 1891a). NHMUK015991484 **A** Mandible, lateral view **B** First and second maxillae, ventral view **C** second maxillary article III and pretarsus, ventral view **D** forcipular segment, ventral view **E** right forcipule, ventral view.

**Figure 9.**
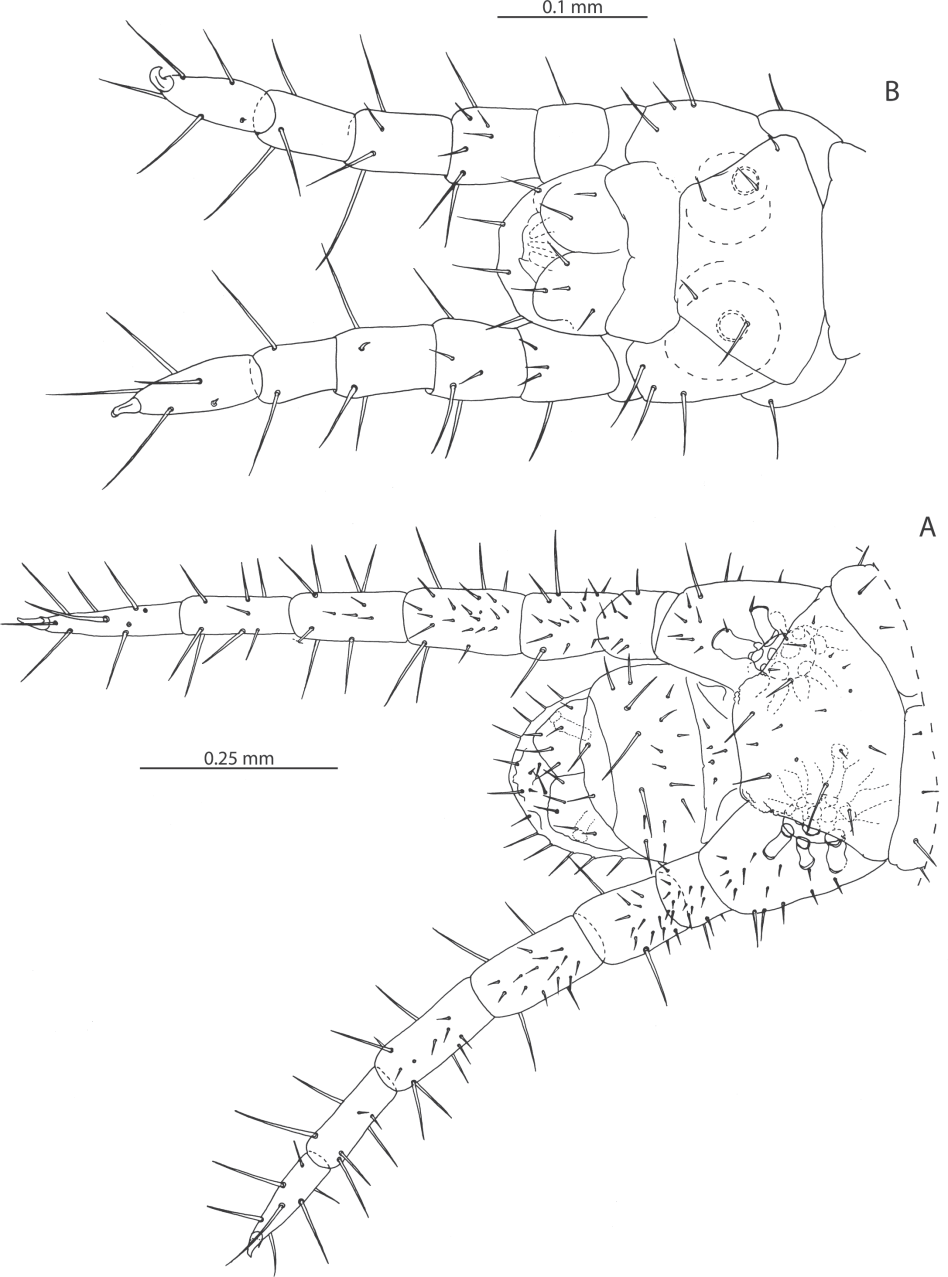
*Tuobasydneyensis* (Pocock, 1891a) **A** NHMUK015991484, female ultimate leg-bearing and postpedal segments, ventral view **B** NHMUK015991478, juvenile ultimate leg-bearing and postpedal segments, ventral view.

**Figure 10.**
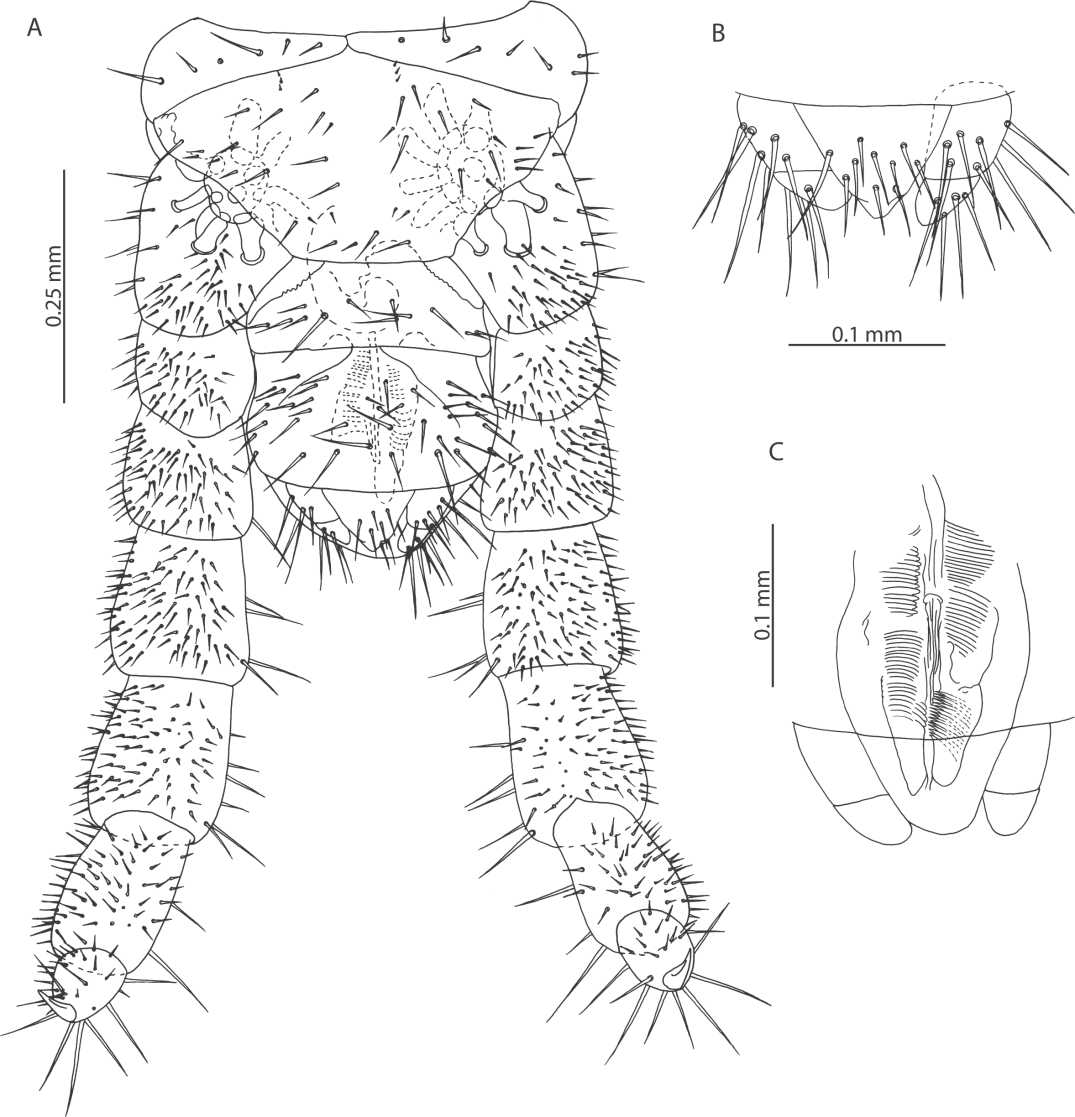
*Tuobasydneyensis* (Pocock, 1891a), NHMUK01591483 **A** male ultimate leg-bearing and postpedal segments, ventral view **B** male gonopods, ventral view **C** penis, ventral view.

Figs 6 , 7 , 8 , 9 , 10

Geophilus (Bothrogeophilus) lemuricus Verhoeff, 1939, syn. nov.
Hovanyx waterloti Lawrence, 1960, syn. nov.

##### Examined material.

19 specimens: NHMUK015991475, 1 specimen, South Island, Aldabra, 13–20.03.1968, leg. B. Cogan & A. Hutson; NHMUK015991476, 1 juvenile, Cinq Cases/Point Hodoul Arga, 27.03.1974, leg. V. W. Spaull; NHMUK015991477, 1♀, inside fallen dead coconut tree, Picard, Aldabra, 23.02.1974, leg. V. W. Spaull; NHMUK015991478, 3♀, 1♂, 1 juvenile, Cinq Cases, Aldabra, 10–17.03.1974, leg. V. W. Spaull; NHMUK015991479, 1♀, *Cyperusligularis* soil and litter, Dune Patates, Aldabra, 05.06.1974, leg. V. W. Spaull; NHMUK015991480, 1♂, 1 incomplete, *Mystroxylon* and *Dracaena* litter, Gionnet, 03.12.1974, leg. V. W. Spaull; NHMUK015991481, 1♀, 1♂, *Casuarina* litter, Picard, Aldabra, 08.05.1974, leg. V. W. Spaull; NHMUK015991482, 1♂, *Pemphis* litter, Dune D’Messe, Grande Terre, 29.01.1975, leg. V. W. Spaull; NHMUK015991483, 2♂, 1♀, *Casuarina*, Picard, 27.12.1974, leg. V. W. Spaull.; NHMUK015991484, 2♀, *Suriana* litter near Point Hodoul, Grande Terre, 22.03.1974, leg. V. W. Spaull; 1 juvenile, *Cocos* litter, Esprit, Aldabra, 14.12.1974, leg. V. W. Spaull.

##### Remarks.

*Tuobasydneyensis* has previously been reported from the Seychelles (Bonato and Minelli 2010). Material presently described from the Aldabra Atoll, as well as specimens collected on Serpent Island (Mauritius) and other islands of the Seychelles (Bonato and Minelli 2010) can be assigned as conspecific on the basis of the low number of leg-bearing segments (41–45), elongation of the antennal articles (Fig. 6A) and in the shape of the ultimate leg-bearing segment metasternite (Jones 1998).

The only other species of *Tuoba* hitherto reported from the East African coast are *Tuobaposeidonis* Verhoeff, 1901 (Zapparoli 1990) and *Tuobasudanensis* Lewis, 1963. The small number of diagnostic characters separating these two species casts doubt on the validity of *T.sudanensis* or on the Somali record of *T.poseidonis*. Examination of additional material from the East African coast would be necessary to elucidate the diversity of *Tuoba* in Northern and Eastern Africa.

Both species can be reliably differentiated from *T.sydneyensis* in the Western Indian Ocean by the larger number of leg-bearing segments (51–53 in *T.sudanensis* compared to 41–45 in *T.sydneyensis*), greater elongation of the telopodal lappets of the first maxillae (30% of the length of the telopodite as illustrated for *T.sudanensis*; compared to minute in *T.sydneyensis* (Fig. 8B)), shape of the carpophagus structure (with a distinct median “hump” in *T.sudanensis*; lacking any “hump” medially in *T.sydneyensis*), point of the midbody transition (sternites 20–22 in *T.poseidonis* and *T.sudanensis*; sternites 14–15 in *T.sydneyensis*) and the shape of the ultimate leg-bearing segment metasternite (1.6 × wider than long as illustrated for male *T.sudanensis*; 1.9 × wider than long in male *T.sydneyensis*, Fig. 10A). The combination of diagnostic characters presented clearly unify the Western Indian Ocean populations of *Tuoba* under one morphospecies, closely matching the description of *T.sydneyensis*, which is distinct from *Tuoba* species recorded in continental Eastern and Northern Africa.

Verhoeff (1939) described Geophilus (Bothrogeophilus) lemuricus from La Ponce, Mauritius and remarked that it is closely related to (“[…] nahe verwandt […]”) *Geophiluscarpophagus* Leach, 1815. Although incomplete, the description provides several characters that allow reliable assignment to *T.sydneyensis*, with which it agrees in number of leg-bearing segments (47 in *G.lemuricus*, 41–45 in *T.sydneyensis* from the Western Indian Ocean), the apical claw of the second maxillae being reduced in size and not overtaking surrounding setae in length (“[...], überragt nicht die Nachbarborsten.”) (Fig. 8B, C), and in the arrangement of the coxal organs on the coxopleuron of the last leg-bearing segment, which are arranged into a rosette opening in a pouch near the edge of the metasternite (“[...] neben dem Endbeinsternit mündet eine Tasche und in diese eine Rosette von Drüsen”) (Figs 9A, 10A). We consider *G.lemuricus* to be a junior subjective synonym of *T.sydneyensis*, which has been subsequently collected from Mauritius (NHMUK015991411).

###### ﻿Family Mecistocephalidae

**Genus *Mecistocephalus* Newport, 1843**

#### ﻿ Mecistocephalus angusticeps

Taxon classificationAnimaliaGeophilomorphaMecistocephalidae

(Ribaut, 1914)

8692BA1F-9EAF-5459-A438-AB09F9D650CD

Fig. 11B

##### Examined material.

11 specimens: NHMUK015991461, 1♂, *Pandanustectorius* soil and litter, Cinq Cases, 24.03.1974, leg. V. W. Spaull; NHMUK015991462, 1♀, 2 km N of Cinq Cases, 11.03.1974, leg. V. W. Spaull; NHMUK015991463, 6 juveniles, *Sideroxylon* litter, Cinq Cases, 10–17.03.1974, leg. V. W. Spaull; NHMUK015991464, 1♀, *Pandanustectorius* soil and litter, Cinq Cases/Point Hodoul, 27.03.1974, leg. V. W. Spaull; NHMUK015991465, 1♂, Gionnet, Aldabra, 03.12.1974, leg. V. W. Spaull; NHMUK015991466, 1♂, Casuarina, Aldabra, 04.02.1974, leg. V. W. Spaull.

##### Remarks.

**Figure 11.**
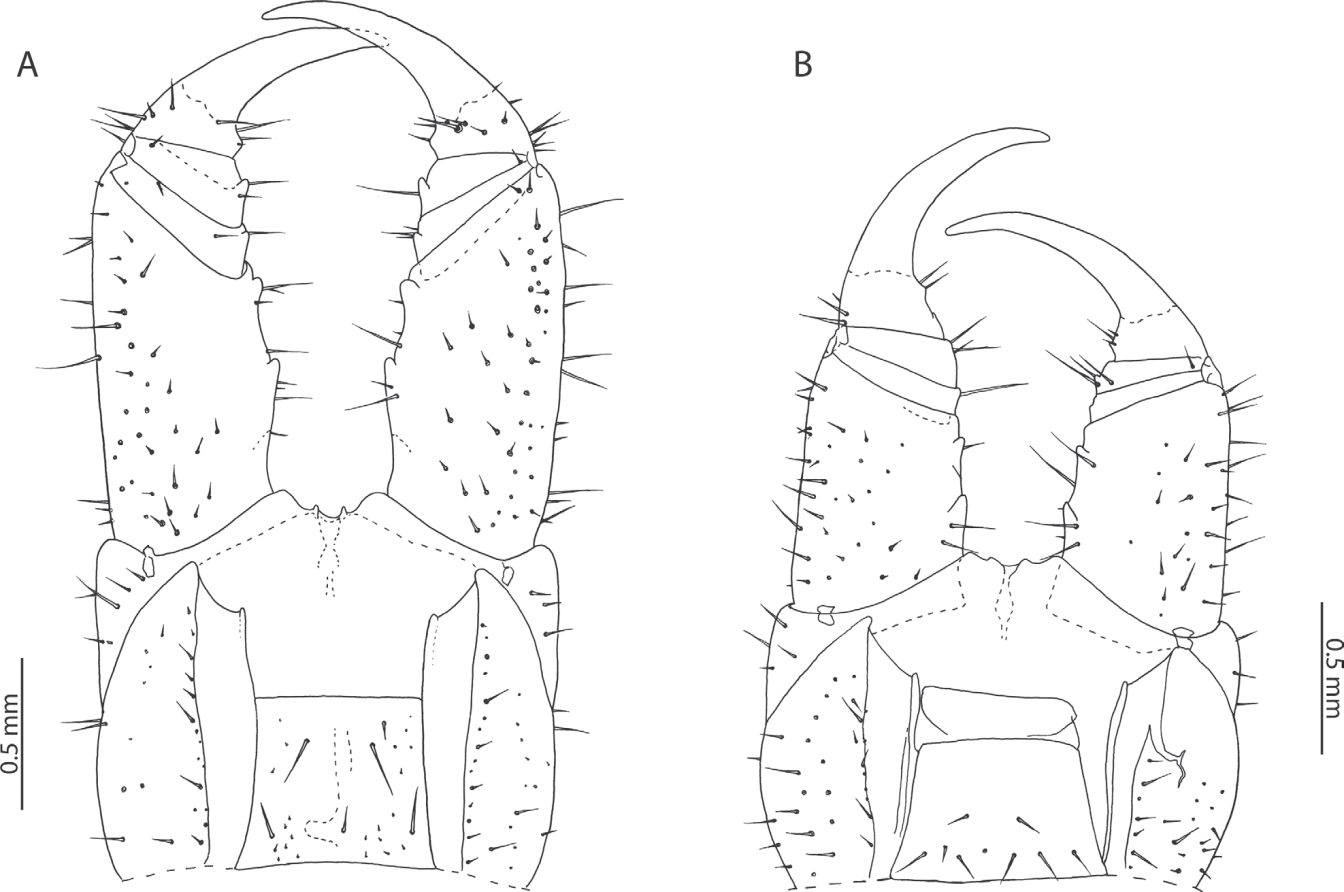
**A***Mecistocephaluslohmanderi* Verhoeff, 1939, NHMUK015991460, forcipular segment, dorsal view **B***Mecistocephalusangusticeps* (Ribaut, 1914), NHMUK015991466, forcipular segment, dorsal view.

Previously recorded from multiple localities near the East African Coast and the Western Indian Ocean (Ribaut 1914; Bonato and Minelli 2010; Popovici et al. 2024), making natural dispersal a likely explanation for the presence of *M.angusticeps* in Aldabra. Presently examined specimens are morphologically indistinguishable from conspecifics recorded from the Seychelles and the Chagos Islands. Although not previously noted, the forcipular cerri were found to be absent in examined specimens (Fig. 11B). In combination with other diagnostic characters (47 leg-bearing segments, conspicuous medial reduction in clypeal reticulation, large distal trochanteroprefemoral denticle and sternal sulcus not furcate), this allows for easy separation from other syntopic *Mecistocephalus* spp. within its range.

#### ﻿ Mecistocephalus lohmanderi

Taxon classificationAnimaliaGeophilomorphaMecistocephalidae

Verhoeff, 1939

7547F35C-6503-5FE1-8A9B-A80624AE48CC

Fig. 11A

##### Examined material.

2 specimens: NHMUK015991459, 1♀, Black Path, Picard, Aldabra, Summer 1975, leg. V. W. Spaull; NHMUK015991460, 1♂, *Casuarina* litter, Picard, 10.12.1974, leg. V. W. Spaull.

##### Remarks.

Originally described from Mauritius (Verhoeff, 1939), *M.lohmanderi* has been found on other Western Indian Ocean Islands (Bonato and Minelli 2010; Popovici et al. 2024). Similarly to *M.angusticeps* (Fig. 11B), examined specimens lack forcipular cerri (Fig. 11A). Presently examined specimens are most similar to *M.lohmanderi* specimens collected from the Diego Garcia atoll (Popovici et al. 2024). Adults (female 34 mm body length, male 34 mm body length) in the Aldabra sample lack dark subcutaneous pigment patches and maintain the characteristic clypeal setation pattern described in *M.lohmanderi* from other localities (Bonato and Minelli 2010; Popovici et al. 2024). Similarly, specimens assigned to *M.insularis* described from Socotra (Lewis and Wranik 1990) match all diagnostic characters outlined for *M.lohmanderi*, and can be referred to this taxon, thus extending its range to island localities in the Northwestern Indian Ocean.

Records of large adult specimens (70–91 mm) assigned to *Mecistocephalusinsularis* from the Arabian Peninsula (Lewis 1996) are fully consistent with the revised description of *Mecistocephalusglabridorsalis* Attems, 1900 from the Seychelles (Bonato and Minelli 2010) and are almost certainly misidentified *M.glabridorsalis*. In particular, the clypeal morphology illustrated for specimens from Saudi Arabia shares the presence of a small non-areolate insula anterior to the plagulae with specimens from the Seychelles and the arrangement of setae in a transverse line on the areolate part of the clypeus. This morphology has hitherto only been recorded in *M.glabridorsalis* and *M.punctifrons* Newport, 1843 (Bonato and Minelli 2004), casting further doubt on the true identity and distribution of *M.insularis*. Insufficient data on morphological variability in *M.lohmanderi* and the uncertain status of *M.insularis* records from past literature prevent further inferences on the taxonomic validity and relationships between these species.

###### ﻿Family Oryidae

**Genus *Orphnaeus* Meinert, 1870**

***Nycternyssa* Crabill, 1959 syn. nov.**

#### ﻿ Orphnaeus dekanius

Taxon classificationAnimaliaGeophilomorphaOryidae

Verhoeff, 1938

2D319CB7-E069-54D0-8FCB-7D9035EF2638

**Figure 12.**
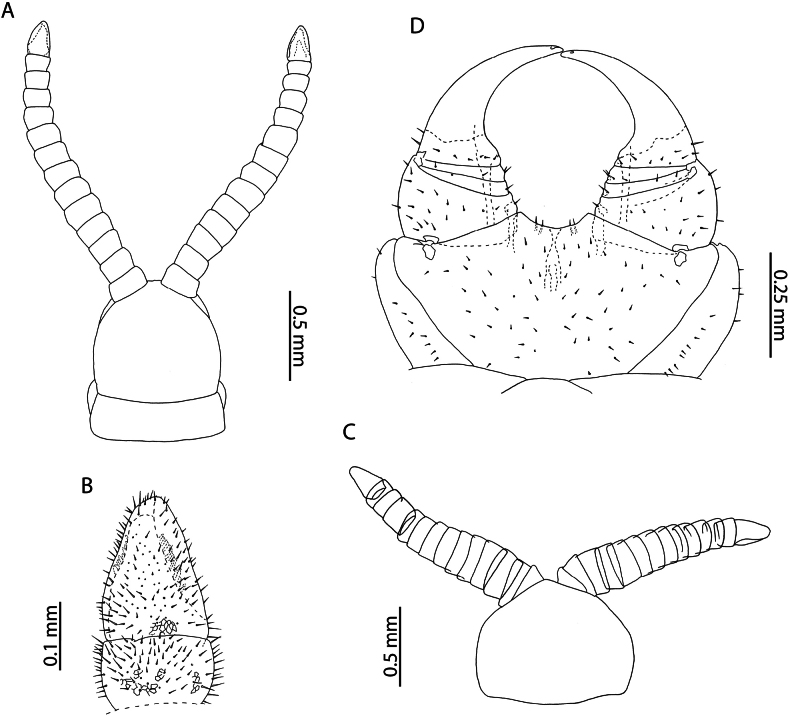
*Orphnaeusdekanius* Verhoeff, 1938 **A, B, D** NHMUK015991470 **A, C** NHMUK015991474. Head and antennae, dorsal view **B** antennal article XIV, dorsal view **D** forcipular segment, ventral view.

**Figure 13.**
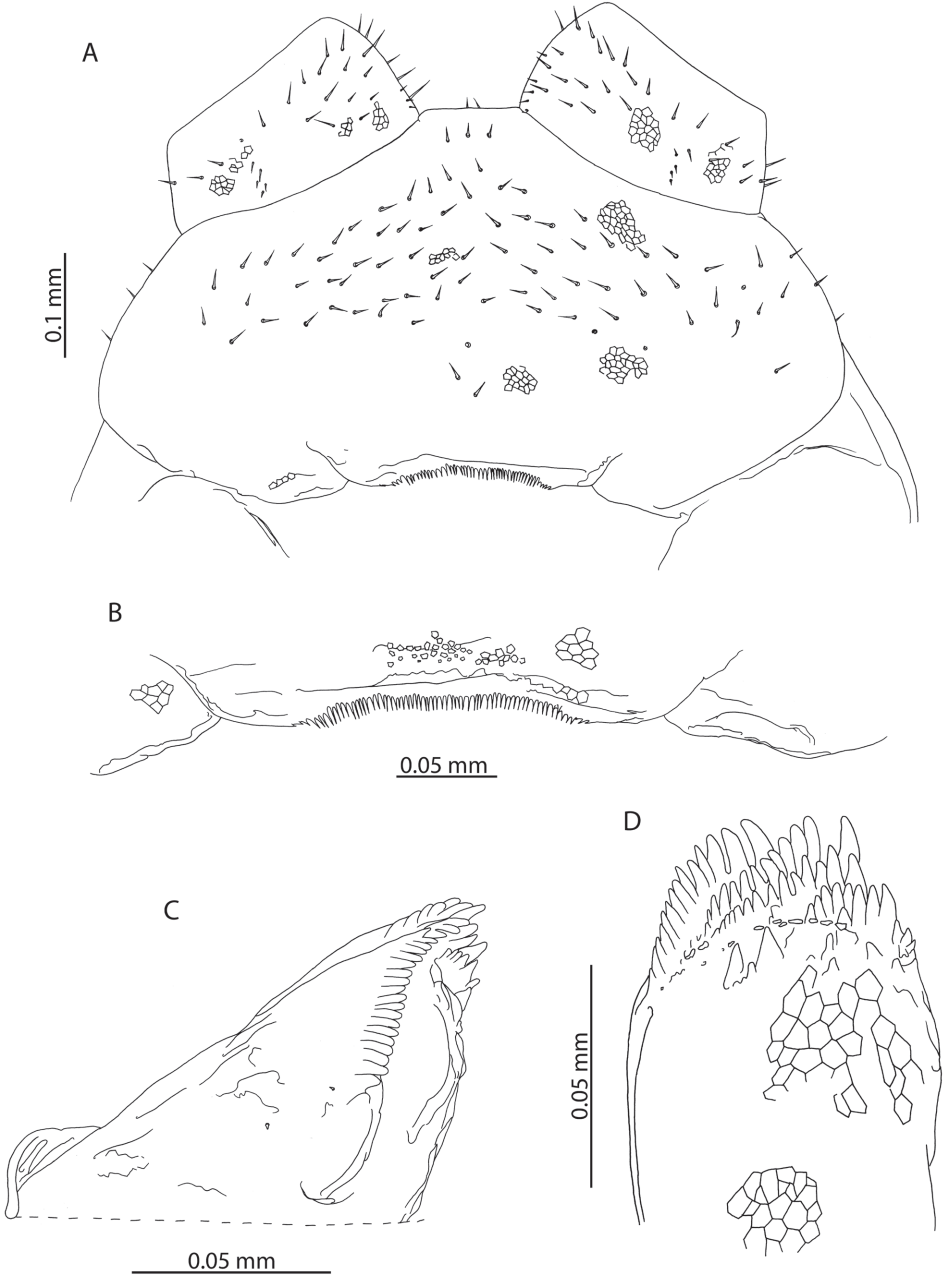
*Orphnaeusdekanius* Verhoeff, 1938. NHMUK015991470 **A** clypeus and labrum, ventral view **B** labrum, ventral view **C, D** Mandible, lateral and dorsal views.

**Figure 14.**
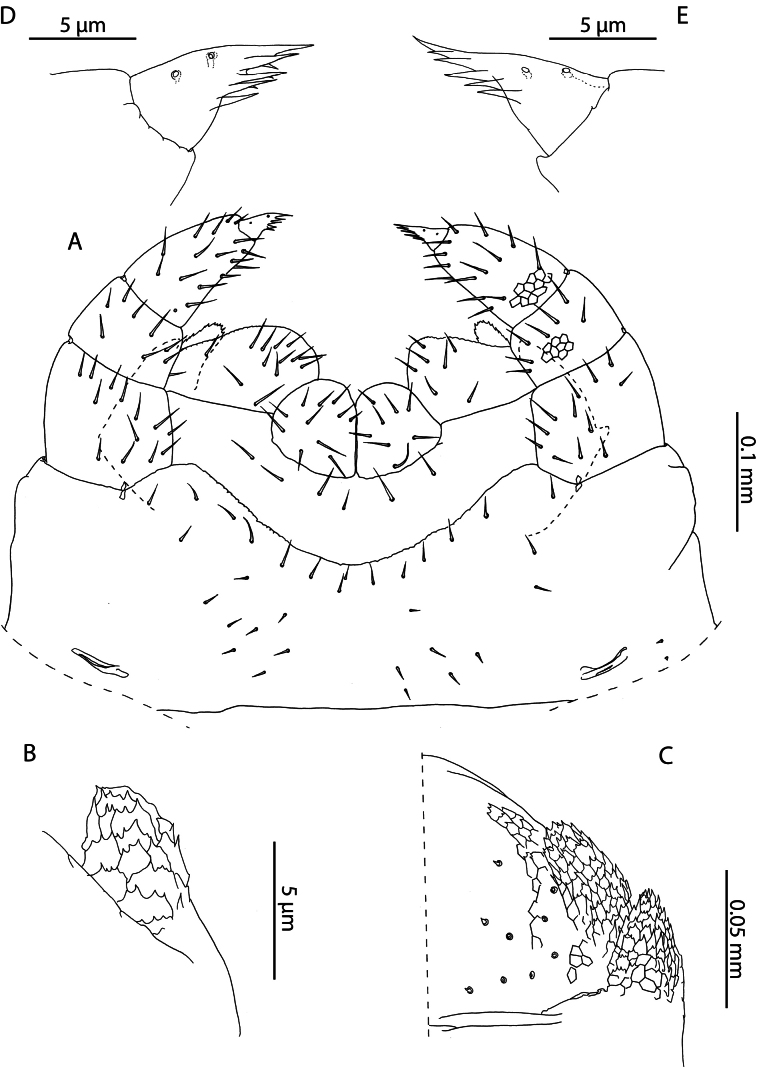
*Orphnaeusdekanius* Verhoeff, 1938 NHMUK015991470 **A** first and second maxillae, ventral view **B** first maxillary right telopodal lappet, ventral view **C** first maxillary left telopodal and coxosternal lappets, dorsal view **D, E** left and right second maxillary pretarsi, ventral view.

**Figure 15.**
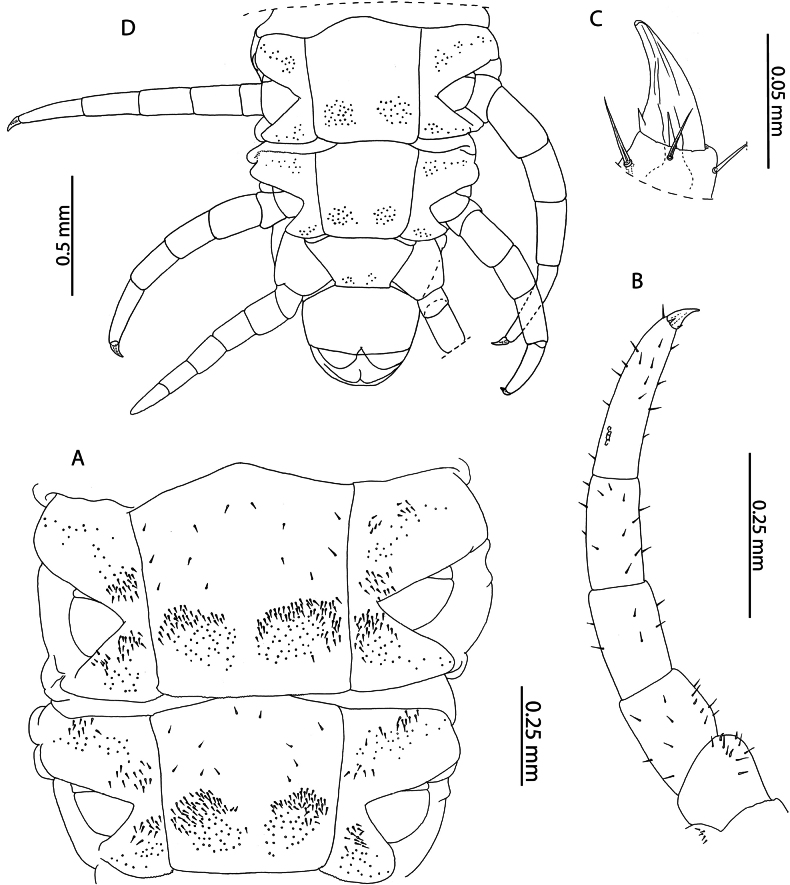
*Orphnaeusdekanius* Verhoeff, 1938 **A, D** NHMUK015991474 **B, C** NHMUK015991470 **A** metasternites of leg-bearing segments 79 – 80, ventral view **B** walking leg, pair 74, lateral view **C** Walking leg pair 74 pretarsus, lateral view **D** Leg-bearing segments 79 – 81, ventral view.

**Figure 16.**
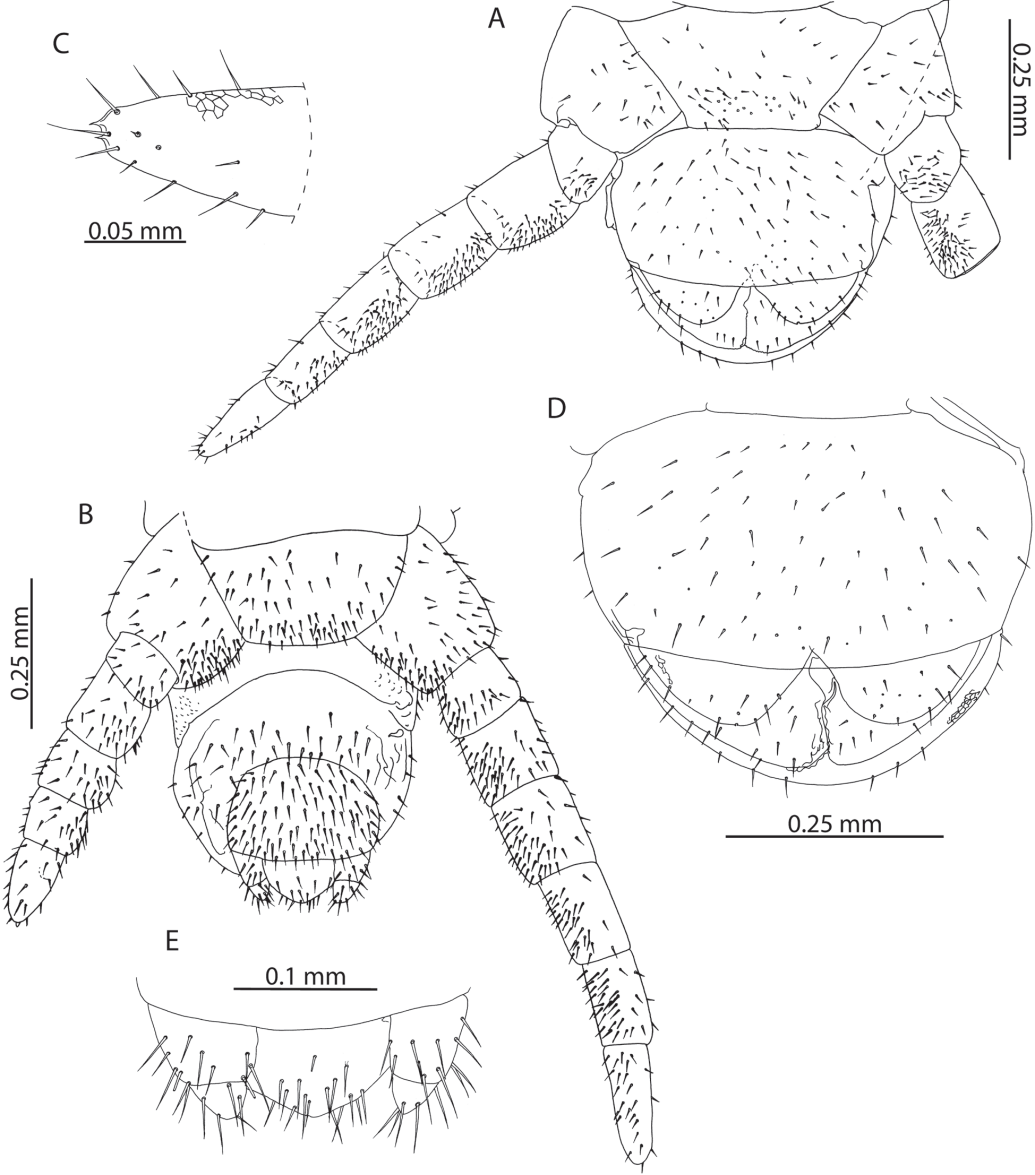
*Orphnaeusdekanius* Verhoeff, 1938 **A, C, D** NHMUK015991471 **B, E** NHMUK015991470 **A, B** ultimate leg-bearing and postpedal segments, ventral view **C** ultimate leg pair metatarsus, lateral view **D** female gonopods, ventral view **E** male gonopods, ventral view.

Figs 12 , 13 , 14 , 15 , 16 , 18D–H

##### Examined material.

6 specimens: NHMUK015991469, 1♂, 40 mm, 73 leg-bearing segments, Picard, Aldabra, 08.10.1974; NHMUK015991470, 1♂, 35 mm, 75 leg-bearing segments, Picard, 09.04.1974; NHMUK015991471, 1♀, 24 mm, 81 leg-bearing segments, Grande Terre, Aldabra, 03.1974, leg. J. Wilson; NHMUK015991472, 1 juvenile, 13 mm, 81 leg-bearing segments, Grande Terre, Aldabra, 05.1974, leg. J. Wilson; NHMUK015991473, 1♂, 37 mm, 75 leg-bearing segments, *Pandanus* litter, Aldabra, 22.03.1974; NHMUK015991474, 1♀, 51 mm, Takamaka (Anse Takamaka), 23–27.02.1968, leg. B. Cogan & A. Hutson.

**Diagnosis.** Medium to large size *Orphnaeus* species, with 73–81 leg-bearing segments and variable but generally present longitudinal bands of dark pigment flanking the central vessel. Mandible with three or four pectinate lamellae. First maxillae with both telopodal and coxosternal lappets present and uniarticulate telopodite. Second maxillary pretarsus spatulate, fringed by acuminate hyaline projections. Posterior trunk metasternites with paired pore fields at the posterior end. Pore fields on posterior metasternites, procoxae and metacoxae bordered by dense groups of setae-like projections. Female gonopods uniarticulate, medially overlapping, with angled, rounded external margin.

##### Description.

***Head and antennae*.** Cephalic plate with broadly rounded anterior margin and straight posterior margin, overlapping the forcipular tergite. Head approximately as broad as long (NHMUK015991473) to 1.2 × broader than long (NHMUK015991474). Antennae approximately 2.5 × longer than head, weakly tapering distally (Fig. 12A). In older specimens, the tapering of antennae and dorsoventral compression of proximal antennal articles are more clearly visible (Fig. 12C). Antennal article XIV 1.9 × longer than the penultimate, with two clusters of sensilla basiconica arranged in lateral pits. Small, spear-like sensilla present at apical end.

***Mandibles*.** Of typical aspect for the genus (Fig. 13C, D). Four conspicuous pectinate lamellae evident, arranged concentrically around distal edge. Proximal to the outermost lamella, isolated projections resembling those on the lamellae are present. Two minute sensilla present laterally.

***Labrum and clypeus*.** Labrum of typical oryid aspect, with short hairlike hyaline projections on middle part (Fig. 13B). Lateral parts incompletely separated from middle part and clypeus by evident sutures. Clypeus with two pairs of postantennal sensilla, a median field of sensilla spanning its mediolateral axis and one pair of prelabral sensilla posterior to these (Fig. 13A). Polygonal reticulation evident.

***Maxillae*.** First maxillae with apically rounded, short coxal projections, bearing 8–11 sensilla (Fig. 14A). Telopodite broadly rounded, uniarticulated, of similar size to the coxal projection, bearing 7–12 sensilla. Both telopodal and coxosternal pairs of lappets present, with distinct spinous reticulation (Fig. 14C). Coxosternal pair of lappets completely obscured by second maxillary telopodite in ventral view. Second maxillary coxosternite with shallow, concave, rounded anterior margin bordered by a row of trichoid sensilla and two groups of trichoid sensilla proximally (Fig. 14A). Metameric pore conspicuous, surrounded by sclerotised rim. Telopodal articles stout. Pretarsus with spine-like projections around entire exterior margin and two pores opening on its dorsal surface (Fig. 14D, E).

***Forcipular segment*.** Forcipular tergite 3.2 × broader than long. Exposed surface of forcipular coxosternite 2.2 × broader than long (Fig. 12D). Chitin lines absent. Anterior margin rounded, deeply concave. Pleural sutures strongly converging posteriorly. Trochanteroprefemur ~ 1.5 × broader than long, with evidently rounded external face. Tarsungulum stout, large, entirely covered by anterior edge of cephalic plate, with smooth inner concavity. Opening of venom gland channel immediately proximal to tip of tarsungulum. All forcipular articles without denticles.

***Trunk*.** Last five or six trunk metasternites with two posteriorly located pore fields (Fig. 15D), anteriorly bordered by dense clusters of hairs (Fig. 15A). All other trunk metasternites with four pore fields, two anterior and two posterior, of equal size on metasternites 2–46, the anterior gradually decreasing in size until disappearing on metasternites 74–76. One single row of paratergites present beginning from the second leg-bearing segment, becoming very conspicuous on the eighth leg-bearing segment. General setation of sclerites sparse.

***Ultimate leg*-*bearing and postpedal segments*.** Ultimate leg-bearing segment metasternite variably trapeziform, 2.3 × broader than long (Fig. 16A, B). Posterior edge with dense field of hairs and occasionally small clusters of pores. Coxopleuron stout, without coxal organs. Telopodite of ultimate leg-pair only moderately inflated in both males (Fig. 16B) and females (Fig. 16A), with dense fields of hairs present on the ventral side of all articles. Pretarsus absent (Fig. 16C). Metatarsus with small hair-like projections at its apical edge.

Female gonopods usually uni-articulated (Figs 15D, 16D), occasionally with an anterior notch or asymmetrical articulation. Lateral edge smooth, rounded, forming strongly acute angle with posterior edge of first genital sternite. Male gonopods biarticulated, bearing 16 setae (Fig. 16E).

##### Remarks.

*Orphnaeusdekanius* was originally described from Trivandrum (Thiruvanathapuram), India (Verhoeff 1938), and maintained as a valid species under *Orphnaeus* until its reassignment to *Nycternyssa*, justified by the description of the female gonopods as “uni-articulate”. A detailed re-evaluation of the status of *Nycternyssa* is provided below.

**Figure 17.**
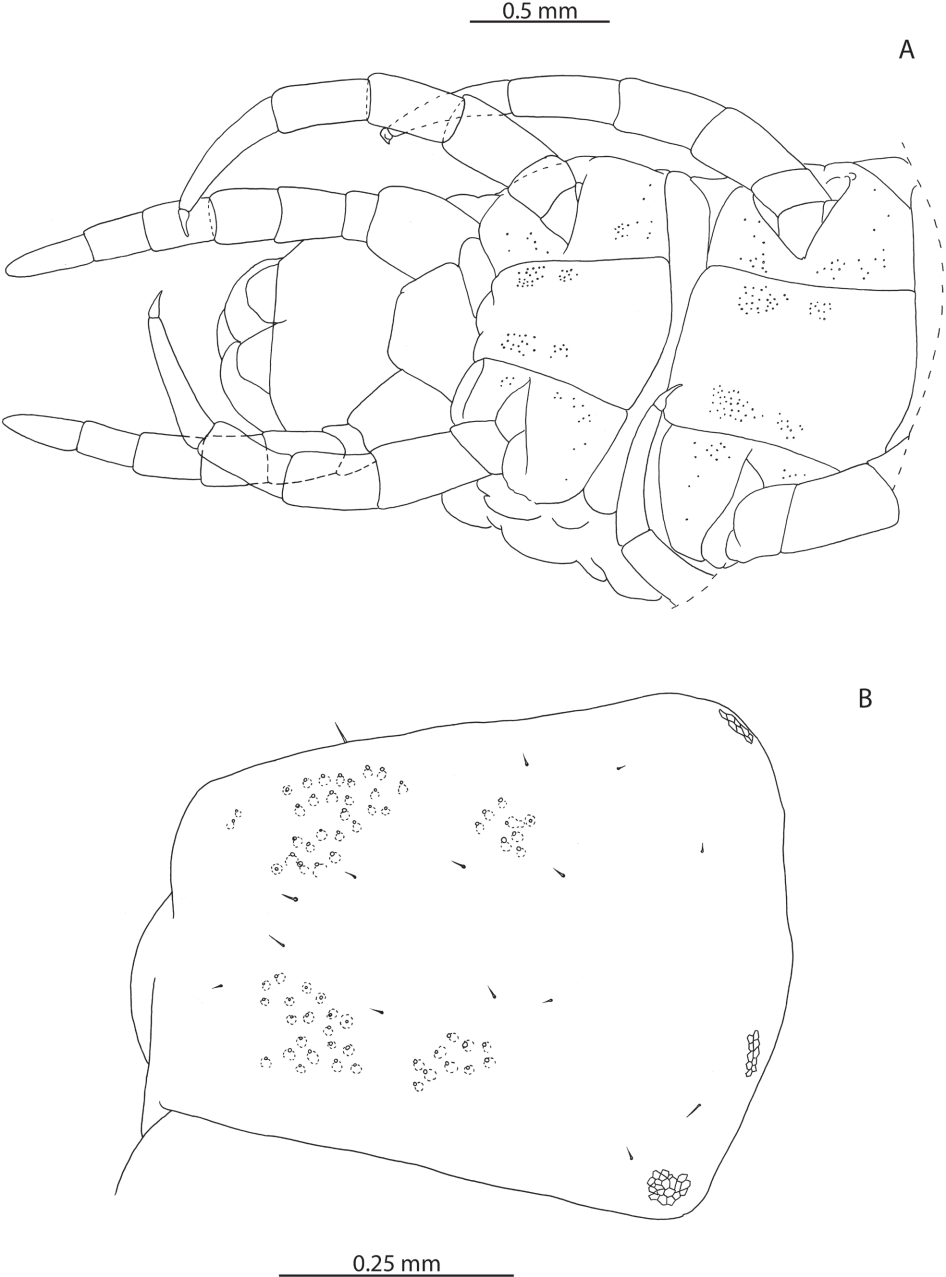
*Orphnaeusbrevilabiatus* (Newport, 1845). NHMUK015991423 **A** leg bearing segments 77 – 79, ventral view **B** metasternite of leg-bearing segment 78, ventral view.

**Figure 18.**
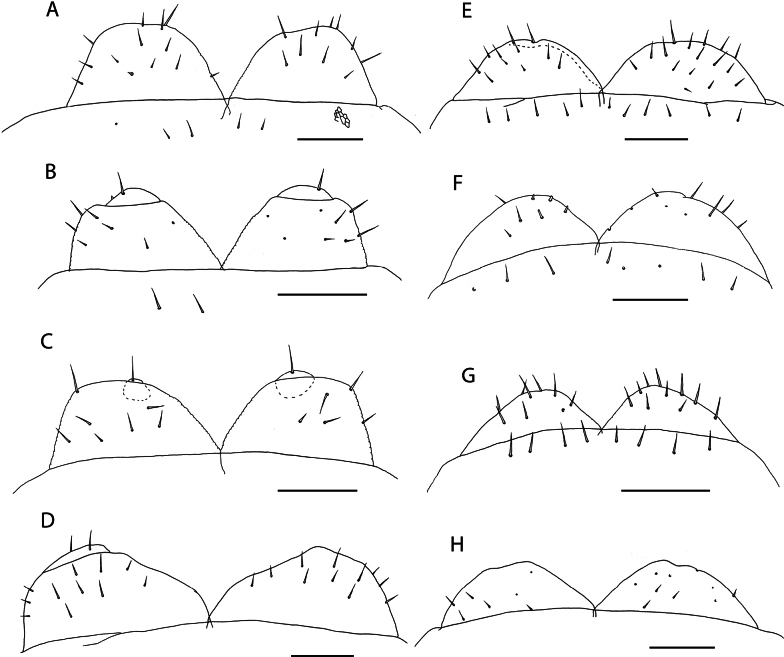
Female gonopods of *Orphnaeus* Meinert, 1870 species **A, B, C***Orphnaeusbrevilabiatus* (Newport, 1845). (A = NHMUK015991423 (Myanmar), B = NHMUK015991421 (Thailand), C = NHMUK015991420 (Singapore)) **D, E, F, G, H***Orphnaeusdekanius* Verhoeff, 1938. (D = NHMUK015991415 (Sri Lanka), E = NHMUK015991413 (Sri Lanka), F = NHMUK015991417 (Singapore), G = NHMUK015991418 (Singapore), H = NHMUK015991416 (Sri Lanka)). Scale bars: 0.1 mm.

Subsequent to its original description, there is no evidence that other specimens had been assigned to either *O.dekanius* or *N.dekania* prior to recent records from the Chagos Archipelago (Popovici et al. 2024). Based on morphology alone, the present specimens are considered conspecific with those collected in the Chagos Archipelago and match the original description of *O.dekanius*. The most salient diagnostic trait allowing for reliable differentiation of *O.dekanius* from *O.brevilabiatus* is the presence of dense clusters of minute setae bordering the posterior pore fields of the former (Fig. 15A). Although this character was illustrated by Verhoeff in the original description of *O.dekanius* (Verhoeff 1938: tafel 8, fig. 61), no mention was made of it in the text of the description. It is unambiguously shared by all specimens studied here from near the type locality of *O.dekanius* (specimens from Sri Lanka listed above under “Specimen data”), and from other localities in the Western Indian Ocean, and is consistent in both sexes in specimens. Setae are sparse in the smallest studied Aldabra specimen (13 mm) but are clustered by a body length of 24 mm. As these clusters of setae are completely absent in specimens identified as *O.brevilabiatus* from near its type locality in Myanmar (Fig. 17) and other localities in mainland and maritime Southeast Asia, we maintain the validity of *O.dekanius* and *O.brevilabiatus* even in light of the variability of female gonopodal articulation in the latter (Fig. 18A–C). The incomplete description of *Orphnaeusmeruinus* Attems, 1909 does not allow for reliable separation from *O.dekanius*, given the inconsistencies in how diagnostic characters for these two taxa are coded in past literature. One salient difference from all *O.dekanius* specimens in our sample is the greatly inflated ultimate leg telopodite in males assigned to *O.meruinus* collected in Oman (Lewis and Gallagher 1993), a character that separates these species even when accounting for body size.

###### ﻿Family Schendylidae

**Genus *Ityphilus* Cook, 1899**

#### ﻿ Ityphilus cf. taeniaformis

Taxon classificationAnimaliaGeophilomorphaSchendylidae

(Lawrence, 1960)

6BD3F1C7-1B37-5025-96C1-8A3015C2A68D

**Figure 19.**
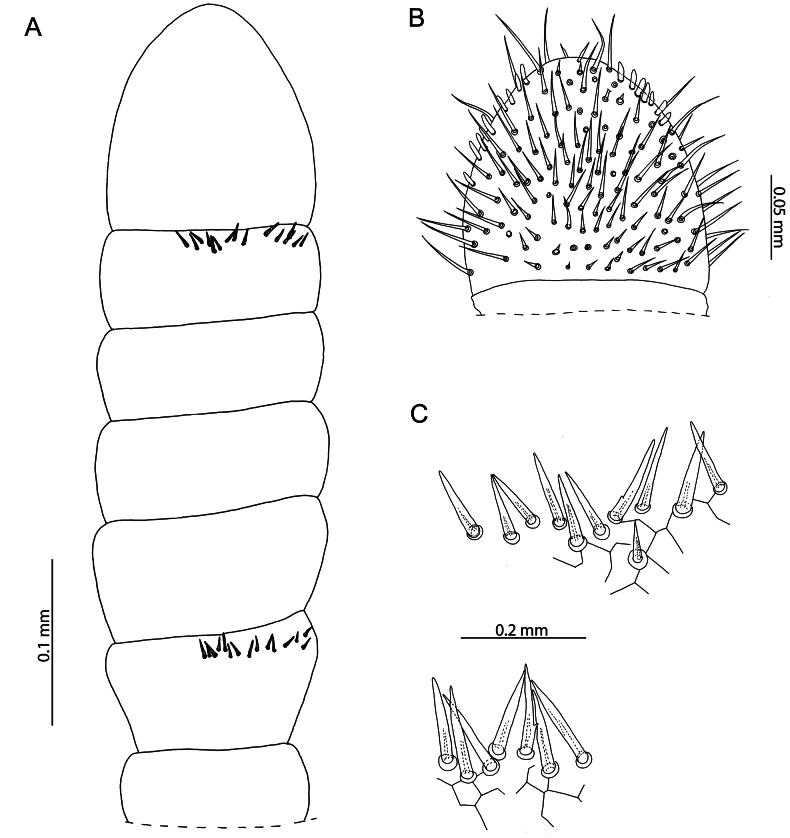
Ityphiluscf.taeniaformis (Lawrence, 1960). NHMUK015991468 **A** right antenna, dorsal view **B** antennal article XIV, ventral view **C** clusters of type c sensilla on antennal article XIII (top) and IX (bottom), dorsal view.

**Figure 20.**
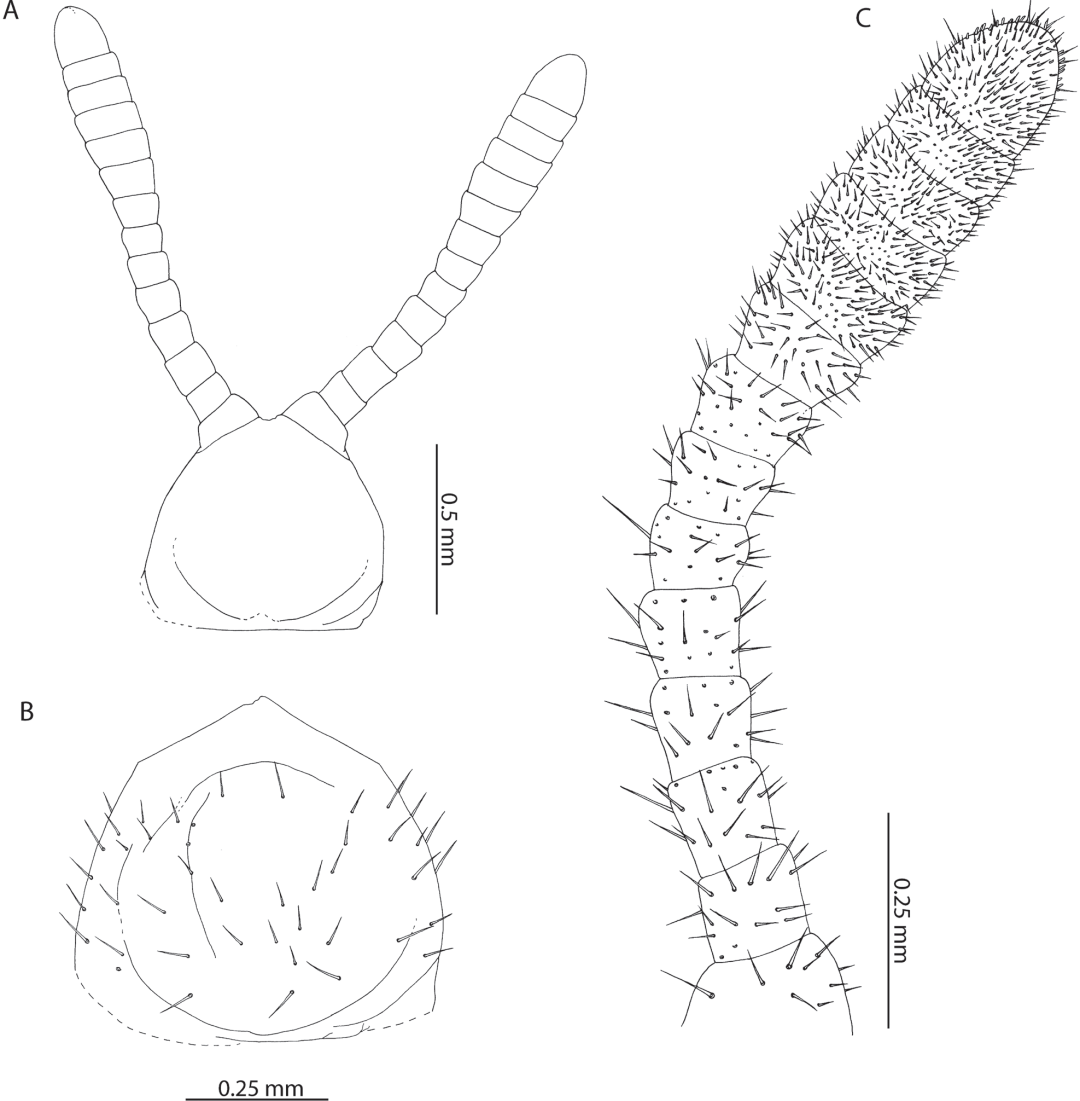
Ityphiluscf.taeniaformis (Lawrence, 1960) NHMUK015991468 **A** head and antennae, dorsal view **B** cephalic shield, dorsal view **C** right antenna, ventral view.

**Figure 21.**
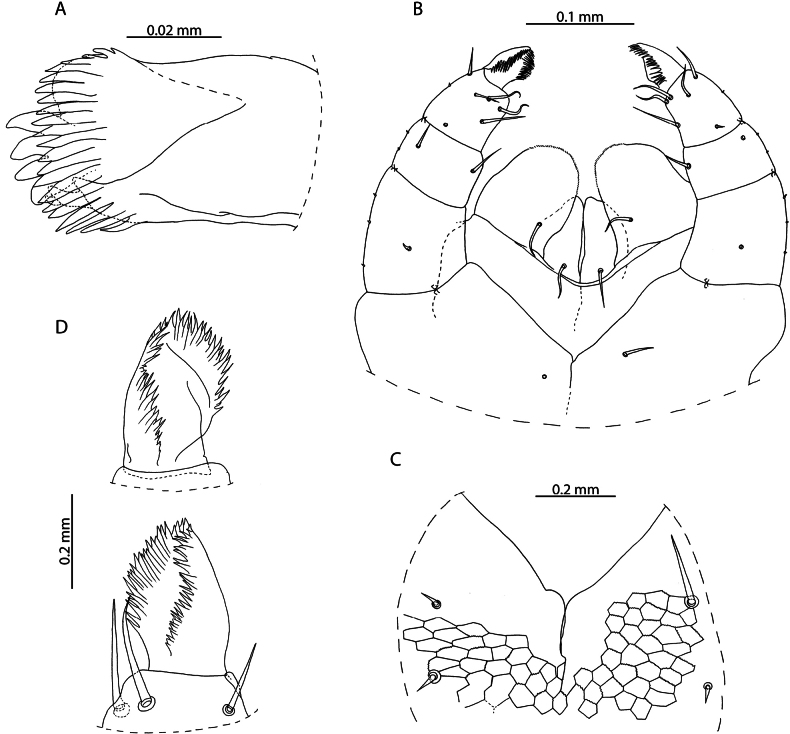
Ityphiluscf.taeniaformis (Lawrence, 1960). NHMUK015991468 **A** mandible, lateral view **B** first and second maxillae, ventral view **C** anterior margin of second maxillary coxosternite, ventral view **D** second maxillary pretarsus left (top), right (bottom), ventral view.

**Figure 22.**
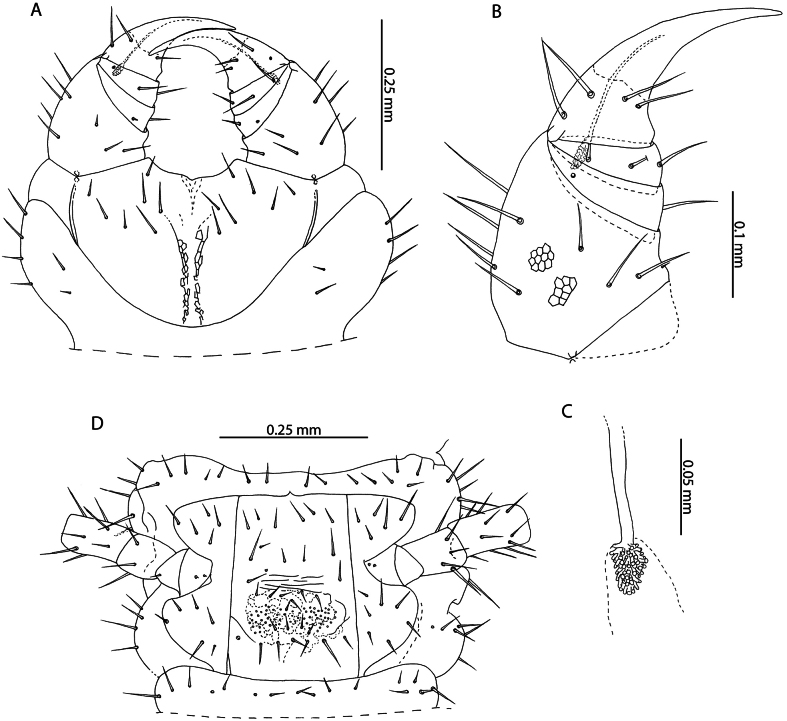
Ityphiluscf.taeniaformis (Lawrence, 1960). NHMUK015991468 **A** forcipular segment, ventral view **B** left forcipule, ventral view **C** calyx of venom gland, ventral view **D** leg-bearing segment 9, ventral view.

**Figure 23.**
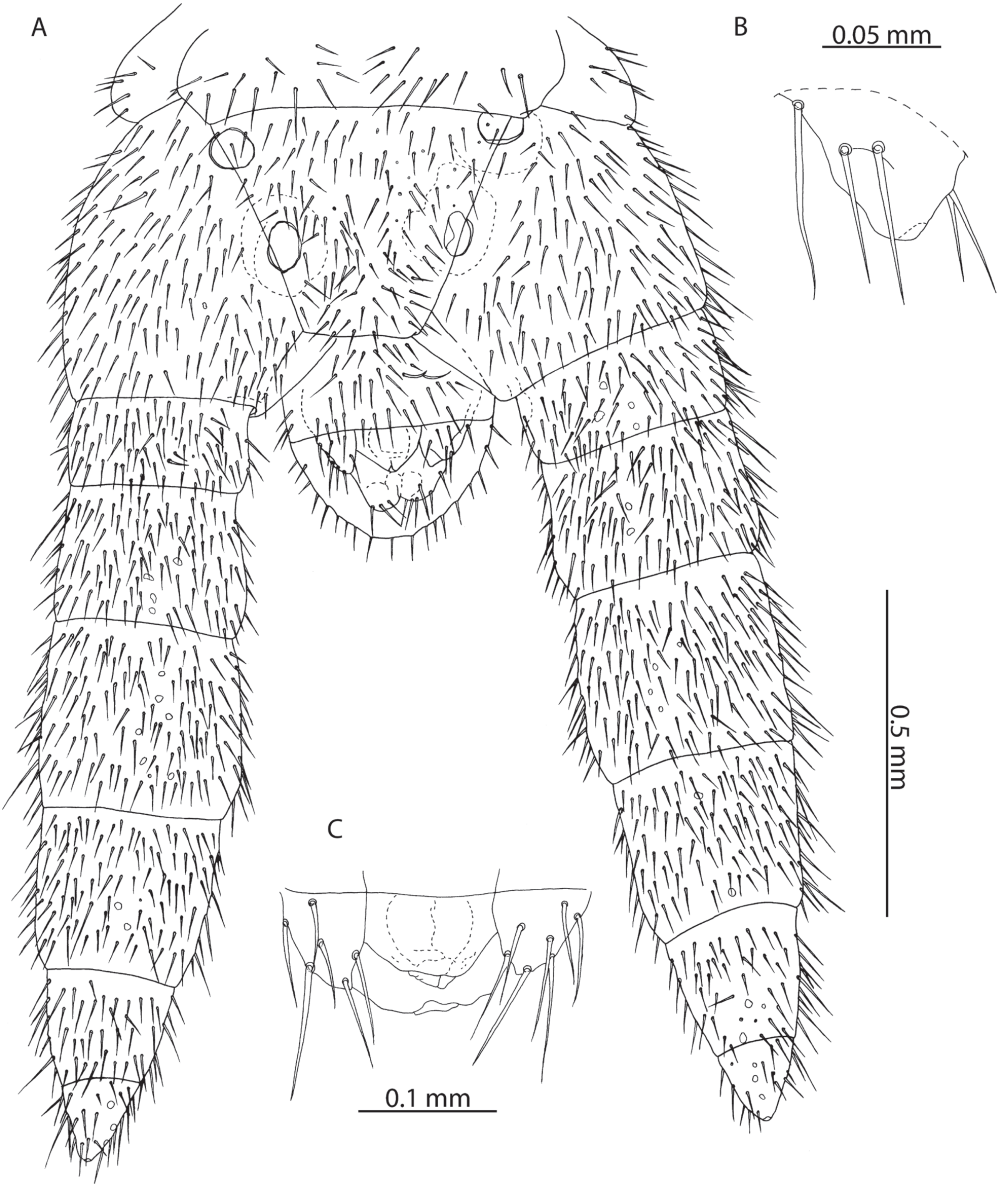
Ityphiluscf.taeniaformis (Lawrence, 1960). NHMUK015991468 **A** ultimate leg-bearing and postpedal segments, ventral view **B** tip of right ultimate leg telopodite, ventral view **C** male gonopods, ventral view.

Figs 19 , 20 , 21 , 22 , 23

##### Examined material.

NHMUK015991468, 1♂, Aldabra, 21.03.1974., V. W. Spaull leg.

##### Description.

***Head and antennae*.** Antennae conspicuously claviform in shape, medially weakly geniculate, with articles IX–XIV widened (Figs 19A, 20A, C). Articles IX and XIII with clusters of type c sensilla (*sensu*Pereira 2017) on the distal edge of the dorsal side (Fig. 19C, D). Article XIV with two lateral clusters of sensilla basiconica and a small number of spear-shaped sensilla at its apex (Fig. 19B). Head approximately as wide as forcipular tergite, 1.14 × broader than long. Curved sulcus near posterior margin. Chaetotaxy of head as in Fig. 20B.

***Mandibles*.** Dentate lamella with seven denticles, only six conspicuous in lateral view. Pectinate lamella with approximately 22 hyaline projections (Fig. 21A).

***Labrum and clypeus*.** Clypeus with a pair of postantennal setae, a cluster of seven medial setae and one prelabral seta. Lateral pieces of labrum narrow, conspicuously sclerotised, lacking any fringes or projections. Medial piece contiguous with clypeus, poorly sclerotised, membranous and lacking conspicuous hairs or projections.

***Maxillae*.** First maxillae with evident, triangular coxal projections, each bearing one sensillum. Telopodites bearing one sensillum each, conspicuously larger than coxal projections and partly covering them (Fig. 21B). Lappets absent. Second maxillary coxosternite with evident but incomplete medial suture, extending for half of its length (Fig. 21C). Each side of the suture bearing one sensillum. Telopodite stout, terminating in large pretarsus. Second maxillary pretarsus spatulate, lateral edges densely fringed (Fig. 21D, E).

***Forcipular segment*.** Exposed face of forcipular coxosternite 2.2 × broader than long (Fig. 22A). Chitin lines present, reaching the condyles. Forcipular trochanteroprefemur 1.25 × longer than broad. Calyx of venom gland elongated, ovoid in shape (Fig. 22C). All forcipular articles without denticles (Fig. 22B). Internal margin of tarsungulum smooth. Extended, forcipules do not reach the anterior margin of the head.

***Trunk*.** 75 leg-bearing segments. Pore fields located on raised areas in the middle of all metasternites excluding those of leg-bearing segments 1, 74, and 75. Shape of pore field oval, medially constricted and anteriorly bordered by a line of setae (Fig. 22D). Colour of pore field bluish grey, conspicuously pigmented relative to surrounding cuticle. Despite the faded colour of the ethanol-preserved specimen, pigmentation of the pore field is conspicuous and the trunk is generally greenish grey in appearance.

***Ultimate leg*-*bearing and postpedal segments*.** Intercalary pleurites separated from ultimate pretergite by evident sutures. Ultimate metasternite trapezoidal, 1.3 × longer than broad. Coxopleura each with two distinct coxal organs, partially covered by the ultimate metasternite (Fig. 23A). Entire ventral side of ultimate leg-bearing segment covered in setae. Ultimate leg telopodite composed of seven articles, all distinctly thickened. Pretarsus absent (Fig. 23B). Metatarsus with a small spine subapically. Intermediate sternite indistinct. First genital sternite with straight posterior margin. Gonopods uni-articulate, flanking penis (Fig. 23C).

##### Remarks.

The taxonomy of *Ityphilus* remains largely unresolved, especially outside of South America, where different authors have disagreed on its relation to *Ballophilus*, alternatively considering it a different genus (Attems 1929) or a synonym of *Ballophilus* (Verhoeff 1939). Recent revision of the genus (Bonato et al. 2007) has maintained the distinction between *Ityphilus* and *Ballophilus*, but remarked on the close relationship between the two, and on cases in which the presence of complete or nearly complete chitin lines is doubtful, such as in *Ityphilusboteltobogensis* (Wang, 1955), despite this character being predominantly used to distinguish the two genera. Similarly, several *Ballophilus* species are described as bearing a cuticular thickening in the usual position of the chitin line (Demange 1963; Pereira et al. 1997), further confounding the utility of this character in taxonomy within the Ballophilidae. The presence of a median sulcus in the second maxillary coxosternite has been shown to be unreliable in separating *Ballophilus* and *Ityphilus*. This character has been described in both *Ballophilus* (Ribaut 1914; Pereira et al. 1997) and *Ityphilus* (Pereira et al. 2000), in some cases as incomplete (Brölemann 1909) (Fig. 21B, C), and is not included in the most recent diagnosis of the latter genus (Bonato et al. 2007).

Verhoeff (1939) described two *Ballophilus* species from Mauritius, *B.lawrencei* Verhoeff, 1939 and *B.mauritianus* Verhoeff, 1939. Both are known from single specimens but, according to their original description, compare closely with *Ballophilusallauadi* Ribaut, 1914 described from Eastern Africa. The Aldabra specimen differs from these species in the presence of pore fields on all but the first and last two leg-bearing segment metasternites (contrasting with the absence of the pore field on the first and the last four metasternites). Additionally, the distal end of the antenna of *B.lawrencei* is illustrated as markedly less clavate than observed for the Aldabra specimen. Despite this, the *Ityphilus* specimen collected in Aldabra overlaps in the shape of the metasternal pore field, its position on a raised area and in the relative elongation of the ultimate leg-bearing segment telopodite and the number of leg-bearing segments with *B.lawrencei*. As all Mauritian *Ballophilus* species are known solely from their holotypes, it is not possible to comment on intraspecific variability that may account for the overlap in these traits.

**Figure 24.**
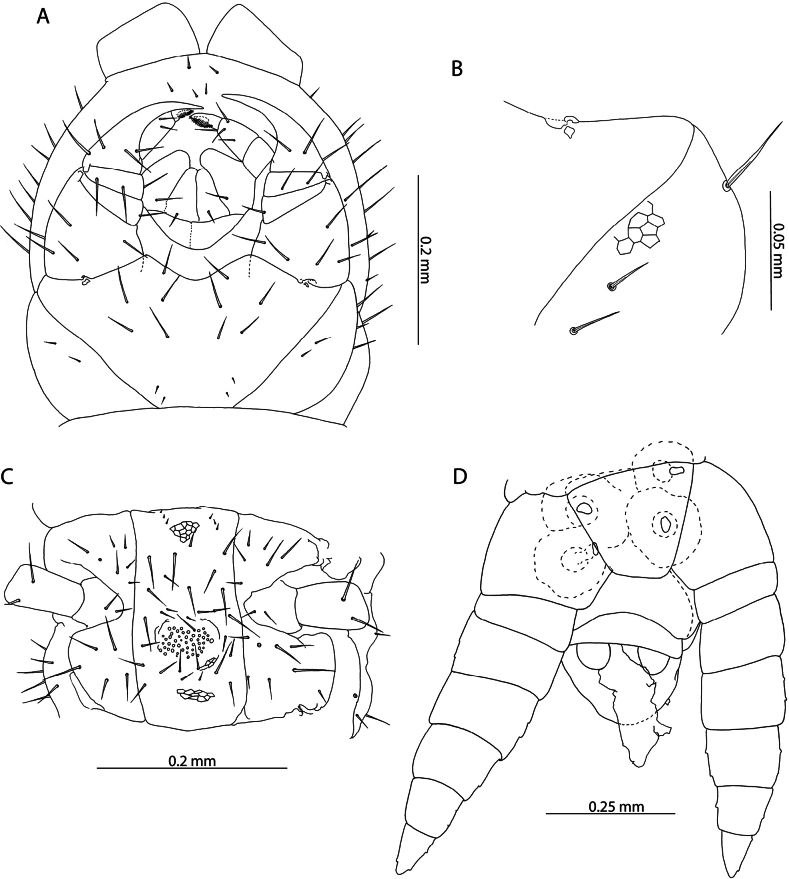
*Ballophilusmaldivensis* Pocock, 1906. BMNH #200555 **A** head and forcipular segment, ventral view **B** magnified view of forcipular coxosternite showing lateral pleurite and condyle, right side, ventral view **C** metasternite of leg-bearing segment 7, ventral view **D** ultimate leg-bearing and postpedal segments, ventral view.

*Ballophilusmaldivensis* Pocock, 1906, described from the Maldives, similarly resembles the Aldabra specimen in the shape of the metasternal pore fields and their pigmentation. The incomplete original description did not allow for comparison of any other putative diagnostic characters beside the number of leg-bearing segments (67 in the female holotype), which is lower than that of the Aldabra specimen (75 in a male). Re-examination of the holotype (Fig. 24), the sole known specimen, revealed several features that further distance it from the Aldabra specimen and bring it closer to the currently accepted diagnosis of *Ballophilus*. *Ballophilusmaldivensis* lacks chitin lines or any cuticular thickenings near their position (Fig. 24A, B). Other important differences from I.cf.taeniaformis from Aldabra include the absence of the metasternal pore field from the last four leg-bearing segments and a more strongly enlarged ultimate leg telopodite (Fig. 24D).

Lawrence (1960) described three species assigned to *Ballophilus* from Madagascar, of which *Ballophilustaeniaformis* Lawrence, 1960 overlaps in nearly all diagnostic traits with the Aldabra material examined, differing only in the number of teeth on the dentate lamella of the mandible (7 in the Aldabra specimen compared to eight or nine in *B.taeniaformis*). Lawrence made no comment on the presence or absence of the chitin line on the forcipular coxosternite for the species he assigned to *Ballophilus*. As noted above, this character has been used to differentiate between the genera *Ballophilus* and *Ityphilus*, although its variability as discussed above and the lack of consensus on the status of *Ityphilus* at the time of Lawrence’s original description of *B.taeniaformis* prompt us to refer the examined specimen from Aldabra to *Ityphilus*.

In nearly all characters examined, the singular specimen from Aldabra agrees with the description of *Ityphilusmelanostigma* (Attems 1900) and the subsequent redescription of this species from specimens collected in the Seychelles (Bonato and Minelli 2010). However, the greatly reduced number of leg-bearing segments (75) relative to the presently known range within *I.melanostigma* (95–101) suggest specific distinction, as extensive variation in leg-bearing segment number is not generally known from other species of *Ityphilus*. Other putative differences to the original description of *I.melanostigma* include the greater relative enlargement of the ultimate leg pair telopodites in the Aldabra specimen, relative to *Ityphilus* specimens illustrated from the Seychelles (Bonato and Minelli 2010).

## ﻿Discussion

### ﻿Composition and affinities of the Aldabran centipede fauna

With the exception of *Australobiusinflatitarsis*, all centipede species previously recorded from Aldabra are represented in the examined sample. This includes nine species new to the atoll, raising the total number of centipede species known from Aldabra to 12. The high abundance of *L.tristani* and *Cryptops* specimens in the examined sample is surprising given the lack of previous mentions of their presence on the atoll.

Of the recorded species, half are shared with other islands of the Seychelles (Lewis 2010b; Stoev and Gerlach 2010) and approximately a third with both Mauritius and the Chagos Archipelago (Table 4; Verhoeff 1939; Lewis and Daszak 1996; Lewis 2002; Popovici et al. 2024). Some have a wide to near-cosmopolitan distribution and have been recorded from mainland localities in East Africa (*Cryptopsnigropictus*, *Mecistocephalusangusticeps*, *Scolopendramorsitans*; Ribaut 1914; Lewis 1969; Zapparoli 1990) and the Indian subcontinent (*Orphnaeusdekanius*, *Scolopendramorsitans*; Verhoeff 1938; Joshi and Karanth 2011) or have been recorded from coastal localities across the entire Indian Ocean (*Orphnaeusdekanius*, *Tuobasydneyensis*; Bonato and Minelli 2010; Popovici et al. 2024). The new records of *S.morsitans* from Aldabra confirm its presence in the outer islands of the Seychelles, which was previously suggested as likely despite the lack of known specimens (Lewis 2007a).

**Table 4.** Occurrence data of centipede species found on the Aldabra Atoll and in nearby areas. Asterisk indicates that morphologically similar congeners have been recorded from the locality but taxonomic revision is required to confirm their identity.

Species	Locality					
	Chagos Archipelago	Madagascar	Maldives	Mauritius	Seychelles (inner islands)	East Africa (mainland)
*Australobiusinflatitarsis*	Not recorded	Not recorded	Not recorded	Not recorded	**Recorded**	Not recorded
*Lamyctestristani*	**Recorded**	**Recorded**	Not recorded	Not recorded*	Not recorded	Not recorded
Cryptopscf.japonicus	Not recorded	Not recorded	Not recorded	Not recorded	Not recorded*	Not recorded
*Cryptopsmauritianus*	Not recorded	Not recorded	Not recorded	**Recorded**	Not recorded	Not recorded
*Cryptopsnigropictus*	Not recorded	Not recorded	Not recorded	**Recorded**	**Recorded**	**Recorded**
*Scolopendramorsitans*	Not recorded	Not recorded	Not recorded	**Recorded**	Not recorded	**Recorded**
Ityphiluscf.taeniaformis	Not recorded	**Recorded**	Not recorded	Not recorded	Not recorded*	Not recorded
*Mecistocephalusangusticeps*	**Recorded**	Not recorded	Not recorded	Not recorded	**Recorded**	**Recorded**
*Mecistocephaluslohmanderi*	**Recorded**	Not recorded*	**Recorded**	**Recorded**	**Recorded**	Not recorded
*Orphnaeusdekanius*	**Recorded**	Not recorded*	**Recorded**	Not recorded	Not recorded	Not recorded*
Ribautiacf.paucipes	Not recorded	Not recorded	Not recorded	Not recorded	**Recorded**	Not recorded
*Tuobasydneyensis*	Not recorded	**Recorded**	Not recorded	**Recorded**	**Recorded**	Not recorded

Comparison with literature data and specimens from Mauritius in the NHM collection reveals that there are no centipedes endemic to Aldabra that are obviously diverging in morphology, and most species occur throughout the Western Indian Ocean islands. Overviews of diagnostic morphological characters given above and in previous surveys of Western Indian Ocean centipedes (Bonato and Minelli 2010; Popovici et al. 2024) strongly suggest that these populations may be closely related and part of a distinctive centipede fauna unique to the Western Indian Ocean, characterised by strong dispersive potential rather than localised radiations within island groups. In the absence of molecular data that would allow for the interrogation of population structure as well as additional vetting of the proposed identifications, and a more complete sample of centipedes from the Comoros and Mascarene island groups, these conclusions are only tentative and based on morphological examination of collected specimens. Similarly, the unresolved taxonomy of the genera *Cryptops*, *Ityphilus*, and *Lamyctes*, particularly in the Western Indian Ocean, makes the apparent absence of *L.tristani* and Ityphiluscf.taeniaformis potentially doubtful from islands in which morphologically similar congeners have been recorded (Bonato and Minelli 2010; Stoev and Gerlach 2010), as future taxonomic revision may reveal that these populations are conspecific.

Human-mediated introduction of centipedes to the Aldabra Atoll is difficult to assess in light of the patchy information on its early human habitation (Stoddart 1971). The atoll was apparently uninhabited when a hydrographic survey was conducted in 1878, and settlement at Picard has been continuous but sparsely populated since 1890. Plantings of coconut, maize, sisal, cotton and other crops by the late 19^th^ Century may account for some soil invertebrate introductions, including centipedes. Approximately 20% of plant species known from Aldabra are considered to be human-mediated introductions, despite a small area devoted to plantations (Renvoize 1971). Human introductions of terrestrial invertebrates are generally poorly characterised across the Western Indian Ocean but have been recorded for the terrestrial gastropod *Achatinafulica* Férussac, 1821 (Peake 1971) and for terrestrial invertebrates in the Chagos Islands (https://chagosinformationportal.org/uploads/Terrestrial_Inverts_of_BIOT_21_03_18_1.xlsx), where 50% of recorded species are listed as introduced and 2.8% as potentially invasive, as well as suggested for the geophilomorph *Tygarrupjavanicus* Attems, 1929 in the granitic inner islands of the Seychelles (Bonato and Minelli 2010).

Being comprised of low to mid-elevation atolls, we consider the Chagos islands a useful guidepost for evaluating the possibility of centipede introductions to Aldabra. The cryptic habits of most centipedes pose challenges to accurately assessing present diversity, let alone introduction potential. One isolated record in Feasibility Study for the Resettlement of the British Indian Ocean Territory Draft Report (https://data.parliament.uk/DepositedPapers/Files/DEP2014-1543/Feasibility_Study_for_the_Resettlement_of_the_BIOT_Draft_Report.pdf) attests to the import of soil from Sri Lanka on some islands, potentially representing a channel for centipede introduction. The morphological similarity of Chagossian *Rhysidalongipes* to Sri Lankan populations of this species (Popovici et al. 2024) well as its classification as a likely invasive (https://chagosinformationportal.org/uploads/Terrestrial_Inverts_of_BIOT_21_03_18_1.xlsx) both indicate that introduction of this species to its Western Indian Ocean locality may have been human-mediated, in association with ballast or imported soil. The lack of any comprehensive historical record of such soil or vegetation transfers on Aldabra makes it difficult to ascribe the presence of any presently reported centipede species to human introduction.

### ﻿The status of *Nycternyssa*

The genus *Nycternyssa* was erected by Crabill (1959), comprising the newly described *Nycternyssastheno* Crabill, 1959 from Okinawa, Japan and four other taxa previously included in *Orphnaeus*, namely *Nycternyssaconspersa* (Verhoeff, 1937a), *Nycternyssadekaniadekania* (Verhoeff, 1938), *Nycternyssadekaniasingaporiensis* (Verhoeff, 1937b) and *Nycternyssaeidmanni* (Verhoeff, 1942). The original generic diagnosis states “*Orphnaeus* differs from *Nycternyssa* in that the female gonopod is divided into two distinct articles while it is simple and undivided in *Nycternyssa*”, with both genera being distinguished from all other related oryids by having only one row of paratergites. No comment was made on characters that differ between male specimens of *Orphnaeus* and *Nycternyssa*. Additional characters listed in the diagnosis of *Nycternyssa*, based on the description of *N.stheno*, are of ambiguous significance or shared by *Orphnaeus*. Crabill (1959) remarked that the first maxillary telopodite is “bipartite”, although the accompanying illustrations provided for *N.stheno* show it as unambiguously uniarticulated and otherwise identical in aspect to illustrations of this structure given for *Orphnaeus* (Fig. 14A). The antennae of *Nycternyssa* are described as barely attenuate and barely flattened proximally, in contrast to the diagnosis of *Orphnaeus* in which the proximal end of the antennae is described as flattened (“platt-gedrückt”) (Attems 1929). This difference of appearance is likely a result of the time since collection and fixation medium of examined specimens, as variation within it can be observed within conspecific individuals from the same locality (Fig. 12A, C), and similar deformations of antennal shape in other geophilomorph taxa have likewise been treated as carrying dubious taxonomic value (Dányi and Tuf 2017; Popovici 2024).

Subsequent mentions of *Orphnaeus* restate the biarticulate nature of the female gonopods as characteristic for the genus (Crabill 1968), or do not comment on this character specifically, but stress the uniarticulate female gonopods of *Nycternyssa* as diagnostic (Bonato 2011). Although this character is regarded as fixed in *Orphnaeus* in the monograph on Geophilomorpha compiled by Attems (1929), several earlier accounts of the type species, *O.brevilabiatus*, do not clearly illustrate biarticulate female gonopods (Saussure and Humbert 1872; Haase 1887), incorrectly identify male specimens as female (Pocock 1889), do not mention the female gonopods in generic keys (Pocock 1893), or illustrate unambiguously uniarticulate female gonopods (Pocock 1896, Saussure and de Zehntner 1902). Although the type material for this species is presumed to be lost, we examined and illustrate the two specimens mentioned by Pocock (1891b) from areas near the type locality in present-day Myanmar (Burma) (Figs 17, 18A–C). One of the two specimens with provenance given as S. Tenasserim is an adult female with unambiguously uniarticulate gonopods (Fig. 18A). Notably Pocock (1891b) mentioned, “I have carefully compared the types of *brevilabiatus* and *lineatus* […]”, implying that the condition of the gonopods in the female specimen from S. Tenasserim and that of the type material did not raise any doubt with respect to the identity of these specimens. As no other morphological characters of the near-topotypic specimens illustrated here suggest they are different from *O.brevilabiatus* as originally described, we consider that the articulation of the female gonopods is likely variable within *Orphnaeus*. Given that there are no characters to support the reciprocal monophyly of both genera, *Nycternyssa* is here placed in junior subjective synonymy.

Specimens identified as *O.brevilabiatus* from other localities displayed variably biarticulate female gonopods, a state previously considered typical for this species (Fig. 18A–C). The present observations rise further doubt on the identity of old records of *O.brevilabiatus* and agree with the reserve of other authors in continuing to treat *O.brevilabiatus* as a single, pantropical species (Würmli 1974). As intraspecific variation in the articulation of the female gonopods is not known in Oryidae, it is possible that the specimens assigned to *O.brevilabiatus* are part of a complex of morphologically similar species.

## Supplementary Material

XML Treatment for Lamyctes tristani

XML Treatment for Cryptops cf. japonicus

XML Treatment for Cryptops mauritianus

XML Treatment for Cryptops nigropictus

XML Treatment for Scolopendra morsitans

XML Treatment for Ribautia cf. paucipes

XML Treatment for Mixophilus

XML Treatment for Tuoba sydneyensis

XML Treatment for Mecistocephalus angusticeps

XML Treatment for Mecistocephalus lohmanderi

XML Treatment for Orphnaeus dekanius

XML Treatment for Ityphilus cf. taeniaformis

## ﻿Additional information

### Author ORCIDs

George Popovici https://orcid.org/0000-0003-0662-7472

Gregory D. Edgecombe https://orcid.org/0000-0002-9591-8011

### Data availability

All of the data that support the findings of this study are available in the main text.
